# Oscillation-Induced Signal Transmission and Gating in Neural Circuits

**DOI:** 10.1371/journal.pcbi.1003940

**Published:** 2014-12-11

**Authors:** Sven Jahnke, Raoul-Martin Memmesheimer, Marc Timme

**Affiliations:** 1 Network Dynamics, Max Planck Institute for Dynamics and Self-Organization (MPIDS), Göttingen, Germany; 2 Bernstein Center for Computational Neuroscience (BCCN), Göttingen, Germany; 3 Institute for Nonlinear Dynamics, Fakultät für Physik, Georg-August Universität Göttingen, Göttingen Germany; 4 Department for Neuroinformatics, Donders Institute, Radboud University, Nijmegen, Netherlands; Glasgow University, United Kingdom

## Abstract

Reliable signal transmission constitutes a key requirement for neural circuit function. The propagation of synchronous pulse packets through recurrent circuits is hypothesized to be one robust form of signal transmission and has been extensively studied in computational and theoretical works. Yet, although external or internally generated oscillations are ubiquitous across neural systems, their influence on such signal propagation is unclear. Here we systematically investigate the impact of oscillations on propagating synchrony. We find that for standard, additive couplings and a net excitatory effect of oscillations, robust propagation of synchrony is enabled in less prominent feed-forward structures than in systems without oscillations. In the presence of non-additive coupling (as mediated by fast dendritic spikes), even balanced oscillatory inputs may enable robust propagation. Here, emerging resonances create complex locking patterns between oscillations and spike synchrony. Interestingly, these resonances make the circuits capable of selecting specific pathways for signal transmission. Oscillations may thus promote reliable transmission and, in co-action with dendritic nonlinearities, provide a mechanism for information processing by selectively gating and routing of signals. Our results are of particular interest for the interpretation of sharp wave/ripple complexes in the hippocampus, where previously learned spike patterns are replayed in conjunction with global high-frequency oscillations. We suggest that the oscillations may serve to stabilize the replay.

## Author Summary

Rhythmic activity in the brain is ubiquitous, its functions are debated. Here we show that it may contribute to the reliable transmission of information within brain areas. We find that its effect is particularly strong if we take nonlinear coupling into account. This experimentally found neuronal property implies that inputs which arrive nearly simultaneously can have a much stronger impact than expected from the sum of their individuals strengths. In such systems, rhythmic activity supports information transmission even if its positive and negative part exactly cancels all the time. Further, the information transmission can adapt to the oscillation frequency to optimally benefit from it. Finally, we show that rhythms with different frequencies may enable or disable communication channels, and are thus suitable for the steering of information flow.

## Introduction

The ground state of cortical networks is characterized by irregular and asynchronous spiking activity [1]–[4] and its dynamics are highly sensitive to perturbations, e.g., missing or additional spikes [2], [3], [5]–[8]. Yet, reliable transmission of information in the presence of such perturbations is assumed to be essential for neural computation. A common hypothesis states that such transmission might be achieved by propagating signals along subnetworks (layers) connected in a feed-forward manner. Indeed, propagation of synchronous and rate signals in feed-forward networks (FFNs) has been demonstrated *in vitro* [9]–[11] and recent experiments suggest that, e.g., the generation of bird-songs relies on activity propagation in feed-forward structures [12]. Moreover, sequential replay observed in hippocampal and neocortical areas also suggest such an underlying feed-forward structure [13]–[18].

Layered feed-forward networks that support propagation of synchrony are termed synfire chains [19]–[23]. The propagated signal is a synchronous pulse-packet [21], [24], i.e., a fraction of synchronously active neurons of one layer which induces synchronous activity in the following, postsynaptic, layer and so on. Robust signal transmission in synfire chains embedded in larger recurrent networks is usually obtained by an increased connectivity (compared to the embedding network) between the neurons of successive layers of the FFN [25]–[27]. Alternatively, increased synaptic efficiencies [28], or the combination of enhanced synaptic weights and non-additive coupling (mediated by fast dendritic spikes, cf. [29]) can enable such a robust propagation [30], [31].

A hallmark of cortical dynamics is the presence of oscillations of various frequencies. A plethora of experimental studies links oscillations in, e.g., delta- (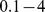 Hz), gamma- (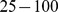 Hz), fast-gamma-band (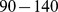 Hz) or the high-frequency range of up to 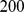 Hz (“ripples”), to attentional states, sensory stimulus selection, ongoing information and memory processing [32]–[40].

In this article we investigate how background oscillations influence the transmission of synchronous activity in feed-forward networks. More precisely, we consider sparse feed-forward structures that emerge as part of a random network and that exhibit moderately enhanced synaptic efficiencies (cf. also [30], [41]). In particular, the feed-forward structures considered are too weak (in the sense of connectivity and coupling strength) to propagate synchronous signals on top of asynchronous background activity. However, we demonstrate that additional oscillatory input, excitatory and inhibitory spike trains generated by an external oscillating neuronal population, can enable robust propagation of synchrony.

We consider both conventional additive couplings, mediated by transient conductance changes on the dendritic input site, and non-additive couplings that take nonlinear processing of inputs by fast dendritic spikes (e.g., [29], [42]–[44]) into account. These dendritic spikes are evoked by highly synchronous inputs (i.e., inputs arriving within a time window of less than a few milliseconds) and cause strong, rapid depolarization in the soma of the postsynaptic neuron, exceeding the depolarization expected from additive processing of inputs. Thereby they may foster directed [30], [31] and undirected [45] propagation of synchrony.

We show that for additively coupled networks, external oscillations support propagation of synchrony only if the (average) excitatory input exceeds the inhibitory input. This exceeding causes a net depolarization of the neurons which in turn enables propagation of synchrony. However, there is no resonance between the propagating synchronous signal and the oscillatory stimulation, and temporally distributed external inputs would have the same effect. In contrast, for non-additively coupled networks the sensitivity of dendritic spike elicitation to synchronous inputs yields resonances to oscillations, i.e., there is a specific stimulation frequency range which enables propagation of synchrony. Dendritic spikes are not suppressed by inhibition [46] such that they support synchrony propagation also if the inputs are balanced, i.e., if the (average) inhibitory input equals (or even exceeds) the excitatory input.

Interestingly, the existence of resonance frequencies provides the possibility to guide synchronous activity along different pathways with distinct resonance frequencies. The mechanism of oscillation-induced signal transmission is robust against changes of the system properties. In particular, networks with peaked and with broad delay distributions exhibit qualitatively similar transmission dynamics. Further, we identify the hippocampus as a core candidate region for oscillation-induced signal transmission as in the hippocampus both high-frequency oscillations and replay of spike patterns are simultaneously observed in experiments.

## Results

**Figure 1:**
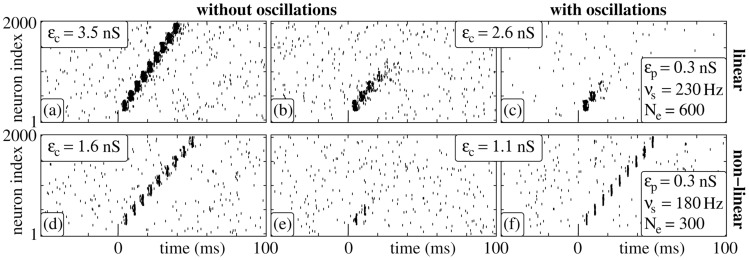
Signal transmission in isolated FFNs (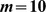, 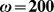, 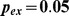) with linear (a–c) and nonlinear (d–f) dendritic interactions. For each dendritic interaction type, raster plots for two different coupling strengths 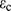 are shown. Panels (a), (b), (d) and (e) display the network activity in the absence of oscillations; in panels (c) and (f) balanced oscillatory input is present (parameters see inset). The stimulation frequency 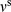 equals the propagation frequency 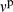 of the stable propagation shown in (a) and (d).

Synchrony propagation through feed-forward structures has been demonstrated for additive and non-additive coupling, and non-oscillatory network background activity [21], [22], [25], [27], [28], [30]. In general, if synaptic coupling is additive (i.e., in the absence of dendritic spikes), the connection strength within the structure, i.e., synaptic efficiencies and/or connectivity, need to be much stronger (perhaps outside the biological plausible range) than for non-additive coupling (cf. Fig. 1a,d and [30], [31]). With too small coupling strength a synchronous signal fails to propagate, the synchronous activity dies out after a small number of layers (Fig. 1b,e).

Interestingly, even balanced oscillatory inputs (cf. Methods Section) may stabilize synchrony propagation if the coupling is non-additive (Fig. 1f), but do not influence or even suppress synchrony propagation in circuits with additive couplings (Fig. 1c).

For too strong couplings, correlations in the spiking of neurons can be amplified over the layers of the feed-forward structure and initiate spontaneous propagation of synchrony (not shown; cf. [47]–[49]). Such spontaneous synchronous spiking can spread over the entire network (if recurrent connections are present), generating a large scale synchronous population burst and a subsequent phase of refractoriness. Throughout the manuscript we refer to network states where large parts of the network spontaneously synchronize like this, as pathological (epileptic-like) activity states. In such states, a meaningful propagation of synchronous signals is not possible: The transmitted signal (induced propagating synchronous pulse) cannot be separated from the background activity (spontaneous synchronous waves).

Whether synchrony propagation is stabilized or enabled depends on features of neurons, network and oscillatory input, e.g., stimulation frequency or synaptic coupling strength. We investigate the mechanism underlying this stabilization numerically and analytically (Supporting Material S1 Text). We identify parameter regions for which synchrony propagation is facilitated by oscillations. In particular, we demonstrate that nonlinearly coupled FFNs show resonance to (balanced and unbalanced) oscillations.

### Synchrony Propagation

As a starting point, we investigate isolated FFNs and briefly describe the mechanism underlying propagation of synchrony in networks with and without dendritic nonlinearities. A detailed description of the neuron and network setup is provided in the Methods Section.

Each neuron of the FFN receives much more input from the external homogeneous background than from the preceding layer. Therefore, in the absence of synchrony, the FFN's dynamics in the ground state is mainly determined by this external background input, and the neurons of the FFN fire asynchronously with a low rate. However, exciting a fraction of neurons of the first layer of the FFN to spike synchronously causes a synchronous input to the second layer, a fraction of which subsequently spikes synchronously. This process continues from layer to layer and thus can induce persistent propagation of synchrony.

One can derive an iterated map (cf. also [30], [31]) that specifies the average number of neurons 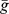 which spike synchronously, i.e., within a certain time interval, given that in the preceding layer 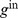 neurons have spiked synchronously. We denote the probability for a neuron in the asynchronous ground state to spike within a time interval of 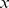 milliseconds after receiving an input of strength 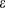 by 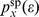. Say that in some layer, 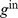 neurons spike synchronously, then each neuron of the following layer will receive some number 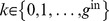 of synchronous inputs of strength 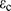. As each of the 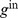 spikes sent is received by every neuron of the postsynaptic layer with probability 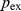, 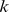 follows a binomial distribution, 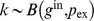, such that on average 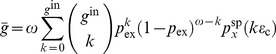
(1)neurons spike within a time interval of 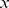 milliseconds.

**Figure 2:**
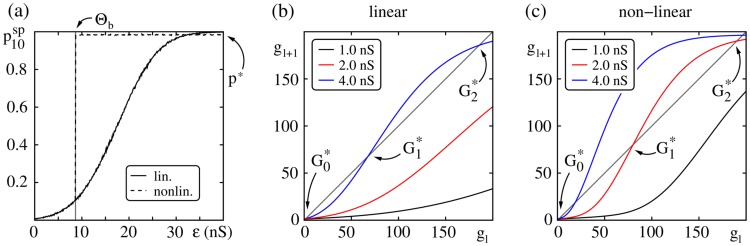
Transition from non-propagating to propagating regime. (a) The probability 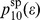 that a single neuron in the ground state (receiving homogenous background inputs) spikes within 10 ms after stimulation by a synchronous input pulse of strength 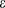. For neurons with linear dendritic interactions (additive coupling; solid line) the spiking probability increases continuously with increasing input 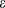. For neurons with nonlinear dendritic interactions (non-additive coupling; dashed line), inputs larger than the dendritic threshold 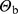 elicit a dendritic spike and therefore the spiking probability jumps to a constant value, 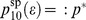, for 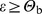. The probabilities are estimated from averaging over 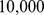 single trials per connection strength. (b,c) Maps (2), specifying the average number of synchronously spiking neurons 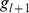 in one layer given that in the previous layer 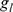 neurons have spiked synchronously; derived from the single neuron response probability in (a) for an isolated FFN (here 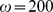, 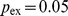). Different colors indicate different strengths of feed-forward connections (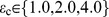
nS); panel (b) shows the map for additive and panel (c) for non-additive coupling. For weak connection strength there is only one fixed point 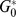 corresponding to the extinction of a synchronous pulse. With increasing coupling strength two additional fixed points 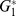 and 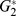 emerge via a tangent bifurcation. This bifurcation marks the transition from a non-propagating to a propagating regime.

We assess the temporal development of the size of the synchronous pulse in every layer by considering 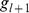 the average number of neurons spiking synchronously in layer 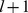 as a function of 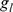 the average number synchronous spiking neurons in the preceding layer 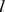. Thus, replacing 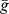 by 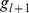 and 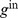 by 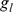 in Equation (1) we obtain the map 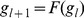
(2)where 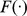 is the continuous interpolation of the right hand-side of Equation (1) for continuous 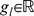. The fixed points of the map (2) determine the stability region for the propagation of synchrony (cf. Fig. 2). For small coupling strength 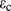, there is only one fixed point 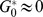 and any synchrony propagation will extinguish within few layers (cf. also Fig. 1b,e). For sufficiently large layer size 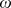 and coupling strengths 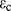, stable propagation of synchrony can be achieved, the size and temporal spread of the synchronous pulse are stable throughout the layers (for an extensive analysis see [31]): This is due to the appearance of two additional fixed points, 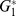 (unstable) and 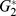 (stable), which emerge via a tangent bifurcation in the map (2) upon increasing 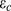. A synchronous pulse 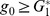 will propagate with a typical group size 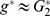.

In a given network, persistent propagation is possible if the connection strengths are larger than some critical value. We denote the critical connection strength, i.e., the bifurcation point at which the fixed points 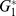 and 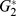 emerge, by 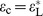 for FFNs with linear dendrites and by 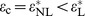 for FFNs with nonlinear dendritic interactions.

Stable propagation of synchrony occurs with a certain propagation frequency 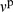, which is defined as the inverse of the average time interval between two consecutive synchronous pulses. 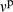 is governed by (i) the synaptic delay and (ii) the the spike latency 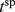, i.e., the average time that an arriving input needs to trigger a spike in the postsynaptic neuron (if it does so). The synaptic delay is fixed for a given setup, but 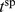 in general depends on the strength of the input and thereby on the connection strength 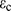.

**Figure 3:**
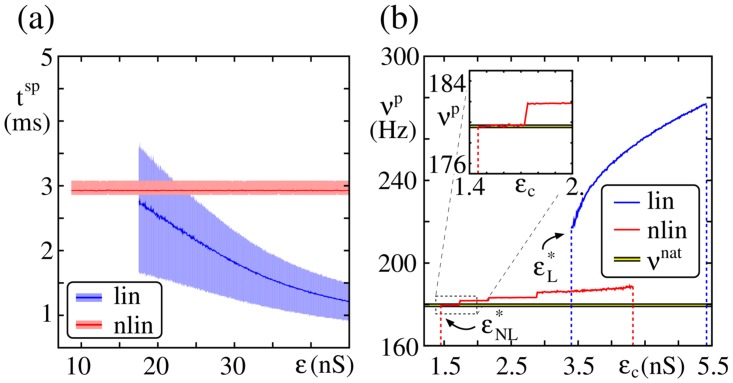
Propagation frequency of a synchronous pulse. (a) Spike latency 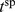 of a neuron after stimulation with an input of strength 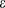 (shaded areas indicate the regions between the 0.2 and 0.8 quantiles; only data for 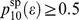 are shown). For neurons with nonlinear dendritic interactions 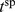 is constant, whereas for neurons with linear dendritic interactions 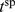 decreases with increasing stimulation strength 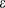. (b) Propagation frequency 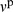 of a synchronous pulse versus strength of the feed-forward connections 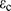 in the absence of external oscillations (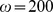, 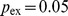); the inset shows a zoomed view of the propagation frequency in FFNs with non-additive couplings for 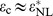. The yellow line indicates the natural propagation frequency 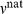.

For networks with linear dendrites, 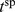 decreases with increasing input strength (cf. Fig. 3a): The increase of the input causes a steeper and steeper rise of the evoked postsynaptic potential, and therefore reduces the (average) time the neuron needs to reach the threshold 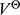. In contrast, 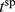 is constant for networks with nonlinear dendritic interactions: The spiking of the neuron is triggered by the additional current pulse mimicking the dendritic spike. This current pulse (and with it the resulting depolarization) is independent of the actual input strength (see also Methods Section), and the rise of the postsynaptic potential is so steep that 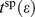 is practically constant for 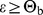. We note that for large input the spike latency 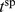 for neurons with nonlinear dendritic interaction is larger than for neurons without: The latency between dendritic stimulation and the onset of the somatic response to the dendritic spike can be estimated to 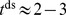
ms [29],[50], and is therefore delayed compared to the onset of the somatic response to the linear (electrically) transmitted signal.

As a consequence of the constancy of the latency 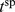, for FFNs with non-additive couplings the propagation frequency 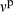 depends only weakly on the connection strength 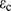. If a propagation of synchrony is enabled for 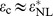, this propagation occurs with a certain ‘natural’ propagation frequency 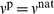. In contrast to linearly coupled FFNs, the propagation frequency remains approximately constant for connection strengths above the critical connection strength, 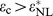 (Fig. 3b). For connection strengths satisfying 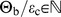 the propagation frequency 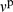 jumps: If 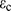 is increased above 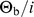 for some 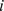, a smaller number 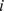 of spikes can trigger a dendritic spike, i.e., a reduced fraction of the synchronous pulse packet is sufficient to trigger dendritic spikes, such that the neurons in each layer tend to spike earlier. This shortens the (average) responding time to the synchronous pulse packet and the propagation frequency increases.

We remark that for large connection strengths 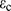, the FFN enters a pathological state of activity: Neurons of one particular layer share inputs from the preceding layer and this causes correlations in their spiking activity. If the single connections become stronger (i.e., only a few inputs are needed to generate a dendritic spike and a somatic output spike) also these correlations become stronger. They may accumulate over the layers of the FFN and lead to spontaneous synchronous spiking activity propagating along the later layers of the FFN [47]–[49]. Thus, there exist cutoff-connection strengths 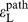 and 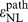 for networks with linear and nonlinear dendritic interactions, above which the global spiking activity is characterized by network oscillations and a meaningful propagation of synchronous activity is not possible anymore.

Whereas signal transmission is possible in FFNs with and without dendritic nonlinearities, the underlying mechanism is different: In linearly coupled networks transmission is achieved by eliciting somatic spikes directly, thus also asynchronous inputs and depolarizing constant external currents may contribute to spike propagation. In nonlinearly coupled networks transmission is mediated by dendritic spikes (all-or-none events), and therefore only highly synchronized spiking input contributes.

### Synchrony propagation in the presence of balanced oscillations

Depending on the coupling strength FFNs may or may not be capable of propagating synchronous signals. But how do external oscillations influence the propagation of synchrony? Do systems with and without dendritic nonlinearities exhibit qualitatively the same behavior?

To answer this question, we first consider isolated FFNs, which receive balanced oscillatory stimulation with frequencies 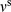 equal to the propagation frequencies 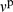 observed for the onset of propagation of synchrony in unstimulated FFNs. Thus we expect the stimulation to be in resonance with the propagating synchronous pulse in the FFN. The impact of different stimulation frequencies and the possibility of complex locking patterns between oscillations and propagating synchrony is investigated in the following Sections.

How does the amplitude of the oscillatory input as controlled by the number 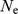 of oscillating (virtual) neurons influences signal propagation?

**Figure 4:**
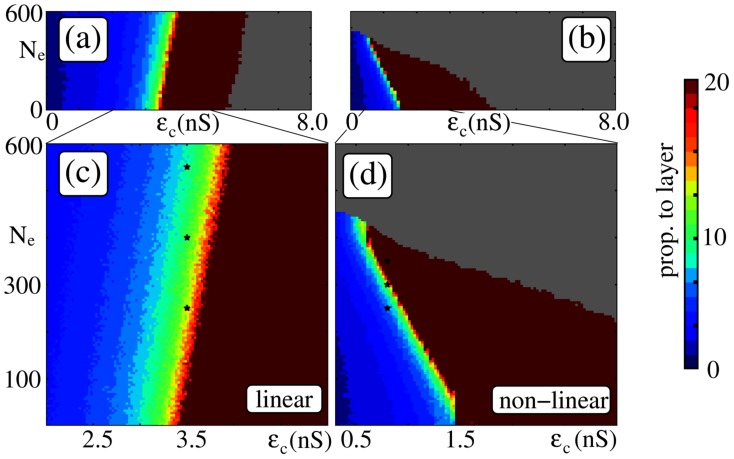
Balanced oscillations can support signal transmission in isolated FFNs (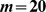, 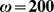, 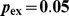). The panels show up to which layer the propagating synchronous pulse (initiated in the first layer in-phase with the external oscillations) is detectable (color-coded) as a function of the coupling strength 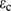 and the amplitude of the external network oscillations, measured by 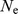. Configurations, where the system enters a pathological activity state (i.e., ongoing spontaneous propagation of synchrony) are marked in gray. Panels (a,c) show simulation results for networks with linear dendritic interactions (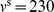
Hz, 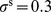
ms) and (b,d) for networks with nonlinear dendritic interactions (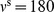
Hz, 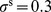
ms); panels (c) and (d) are close up views of (a) and (b). The black stars indicate the values of 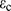 and 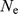 used in Fig. 6a,c. Whereas balanced oscillations hinder signal propagation in additively coupled networks (i.e., require compensation by stronger coupling), they can support it in non-additively coupled ones. Other parameters are 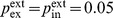, 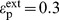
nS, 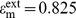
nS.

For networks with additive couplings we find that the critical connection strength (i.e., the minimal connection strength which enables propagation of synchrony) increases with increasing oscillation amplitude 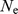 (details on the setup of the oscillatory input are provided in the Methods Section) as illustrated in Fig. 4a,c: The additional input is balanced, so that the mean input to each neuron is constant (for all 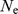), but both the mean excitatory and inhibitory conductances are increased. In this high-conductance state the effective membrane time constant decreases and consequently the amplitude and the width of postsynaptic potentials decrease [51], [52]. In other words, the additional inputs arising from oscillations decrease the excitability of the neurons. Thus, stronger inputs (in terms of conductances) are needed to generate the same depolarization as in networks without external oscillations and the critical connectivity, 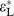, increases. This is the same phenomenon that hinders synfire-explosions [26], [53] in networks with conductance-based synapses as described in [27].

In contrast, in networks with non-additive couplings, the critical connection strength decreases with increasing oscillation amplitude 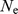 (see Fig. 4b,d). In such networks the propagation of synchrony is mainly mediated by dendritic spikes. Dendritic spikes are elicited if the excitatory input on a dendrite within a certain time-window, 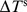, is larger than the dendritic threshold 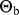. Inhibition fails to suppress dendritic spikes [46] and thus its increase does not hinder signal propagation. If the frequency 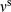 of network oscillations is in the range of the natural propagation frequency 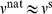, and the oscillations are in phase with the propagating signal, the synchronous pulse from the preceding group arrives at each layer synchronously with the oscillatory inputs. Thus, less input from the preceding layer is needed to reach the dendritic threshold. Taken together, by effectively lowering the dendritic threshold 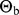 the external inputs reduce the critical connectivity 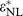. In Fig. 4b,d we show that this reduction can yield propagation of synchrony at drastically reduced synaptic efficiencies within the FFN; in the given example the critical connection strength 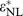 is reduced by a factor of two to three (from 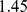
nS to 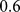
nS).

The downside of the robustness of dendritic spikes to inhibition is that even balanced oscillations may cause pathological activity if oscillation amplitude becomes too strong: With increasing amplitude 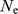 the neurons of the FFN become more and more sensitive to inputs from the previous layer. Thus, similar to the regime of overly strong feed-forward connections, correlations in their spiking activity accumulate along the layers of the FFN [47]–[49] and induce spontaneous propagation of synchrony (gray areas in Fig. 4).

### Synchrony propagation in the presence of unbalanced oscillations

Like balanced oscillations also unbalanced oscillations may be expected to alter the propagation efficiencies of FFNs: The average external excitatory input is larger or smaller than the inhibitory input, and thus the average ground state membrane potential of the neurons is shifted which influences the neurons' excitability. As we show below this shift clearly influences propagation of synchrony in additively coupled networks, but has only a weak effect in non-additively coupled systems.

**Figure 5:**
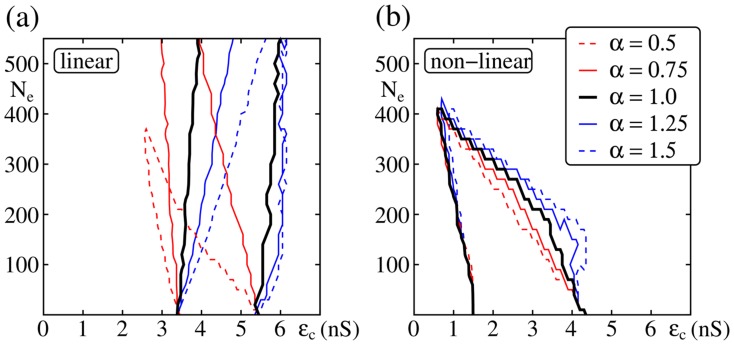
Support of propagation of synchrony by unbalanced oscillations. Same setup as in Fig. 4, but with altered inhibitory coupling strength 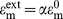 as indicated in (b). The lines inclose the parameter regions for which an initial synchronous pulse is detectable up to the final layer. (a) For FFNs with linear dendritic interactions unbalanced oscillations may foster propagation of synchrony, if the excitation exceeds the inhibition (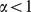, i.e., 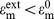; red lines) or impede it, if the inhibition exceeds the excitation, respectively (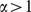, i.e., 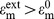; blue lines). (b) In contrast, in FFNs with nonlinear dendritic interactions the balance between excitation and inhibition has only a weak effect on the parameter region in which robust propagation of synchrony is possible.

For a given excitatory coupling strength 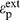 we denote the corresponding balanced inhibitory coupling strength by 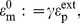
(3)where 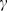 is chosen such that the peaks of the excitatory and inhibitory postsynaptic potentials are of equal amplitude when the input is received at resting potential (cf. also Methods Section). We consider isolated FFNs stimulated by oscillations as in the previous section, but we decrease or increase the strength of the inhibitory inputs by a factor 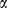 compared to the balanced regime, i.e.,



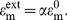
(4)For additively coupled networks and 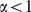 such input indeed promotes synchrony propagation (cf. Fig. 5a, red lines): The oscillatory input depolarizes the neurons of the FFN and thus less synaptic input is needed to elicit a somatic spike; the critical connectivity 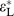 decreases. At the same time, the increased excitability of the neurons lowers the threshold for pathological activity, 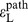. Likewise, for 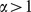 the neurons are hyperpolarized by the oscillatory input which impedes the generation of somatic spikes; the critical connectivity 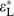 increases (cf. Fig. 5a, blue lines).

In contrast, in non-additively coupled networks, the critical connectivity 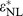 is largely unaffected by changing the balance of inhibition and excitation (cf. Fig. 5b). Here, propagation of synchrony is mediated mainly by dendritic spikes, and their generation is not influenced by inhibition. Pathological activity is induced if correlations in spontaneous spiking activity accumulate over the layers. Because inhibition reduces the overall spiking activity (and also the probability that a dendritic spike triggers a somatic one), with increasing 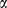 (and thus increasing inhibition) the pathological threshold 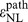 increases.

We note that although unbalanced oscillations may promote propagation of synchrony in additively coupled networks, the mechanism underlying this support differs from propagation of synchrony in non-additively coupled networks. The effect is attributed to the increase of the (average) ground state membrane potential and, as we demonstrate below could as well be obtained by additional constant (over time) input currents with the same strength as the mean input due to the oscillations.

### Network Resonance

Oscillations may support propagation of synchrony (if in resonance), but how does their actual impact depends on system features such as frequency and amplitude of external oscillations? In the following, we investigate which frequency ranges support or hinder synchrony propagation. In particular, we show that networks with non-additive coupling exhibit resonance to stimulations where the frequency 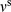 is rationally related to the natural propagation frequency 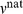. In networks with additive couplings, we do not find such a resonance effect, even if the stimulation is unbalanced and therefore supports signal propagation.

**Figure 6:**
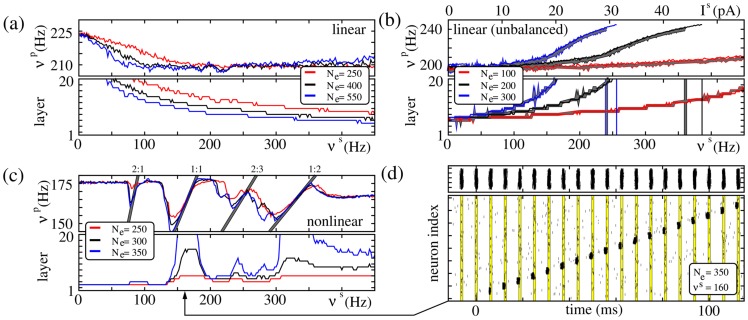
FFNs with nonlinear dendritic interactions show resonance. Same network setup as in Fig. 4; coupling strengths are (a) 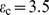
nS, (b) 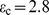
nS and (c,d) 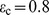
nS. (a–c) The upper panels display the propagation frequency 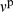 of the synchronous signal, the lower panels show the layer up to which propagation occurs, as a function of the stimulation frequency 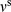 for FFNs with (a,b) linear and (c) nonlinear dendritic interactions. Different colors represent different amplitudes 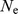 of external oscillations as indicated by insets. In additively coupled FFNs (a) balanced oscillations hinder synchrony propagation, whereas (b) unbalanced oscillations (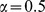, i.e., excitation exceeds inhibition, cf. Equation 4) support it. This support, however, might be equally well achieved by temporally constant additional excitatory inputs: The thick gray filled lines indicate the propagation properties of an FFN, where single neurons receive constant additional current 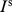 (red; upper vertical axis), 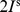 (black) or 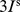
(blue). For very strong depolarization (high 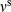 or 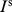) the network enters a pathological activity state; this break-down of network stability is indicated by the vertical lines in the lower panel. In non-additively coupled FFNs even (c) balanced oscillations foster synchrony propagation and, in contrast to additively coupled FFNs, the propagating signal may lock to the oscillatory stimulation if the ratio 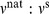 is rational; the gray lines indicate 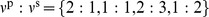. This locking is illustrated in (d): Raster plots of spikes of the external oscillating population (upper panel) and of the FFN (lower panel). The yellow lines indicate the time intervals 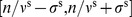 for 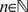, containing 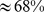 of the spikes of the external oscillatory population (cf. also Fig. 7).

First, we consider networks with linear couplings: As pointed out in the previous section, balanced oscillatory inputs decrease the excitability of the neurons of the FFN. Thereby it decreases the capability of the network to propagate synchronous signals for all stimulation frequencies 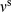. With increasing 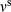, the total number of input spikes per unit time increases and the effective time constant decreases further such that the propagation becomes more and more difficult. Fig. 6a illustrates that the presence of balanced oscillations indeed inhibits synchrony propagation increasingly, the stronger and the more prominent the oscillations are (i.e., larger 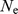 and 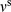).

The support of signal transmission by unbalanced input (cf. Fig. 5) is caused by an increase of the ground state's membrane potential. With increasing 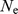 and 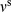 this depolarization increases (increased net excitation) and thus facilitates synchrony propagation more and more. Likewise, the propagation frequency 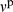 increases until the stimulation gets too strong and the system enters a pathological activity state. We do not observe resonance to the oscillatory stimulation, and the promotion of propagation of synchrony can equally well be obtained by an additional constant (over time) excitatory input 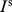 which is proportional to the stimulation frequency 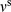 (cf. Fig. 6b).

In contrast, networks with non-additive couplings show resonance, and even balanced oscillations enable propagation of synchrony for configurations where signal propagation fails for homogeneous external background (i.e., in the absence of external oscillations, cf. Fig. 4b,d). For stimulation frequencies 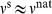, we observe a locking of the propagating signal to the external stimulus: The input from a preceding layer is not sufficient to excite sufficiently many neurons to spike synchronously and to enable persistent propagation. It can, however, take place if there is additional input. An oscillatory external input then influences the timing of the propagating pulse-packet and the propagation frequency 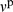 locks to the stimulation frequency 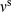 (cf. Fig. 6c,d).

**Figure 7:**
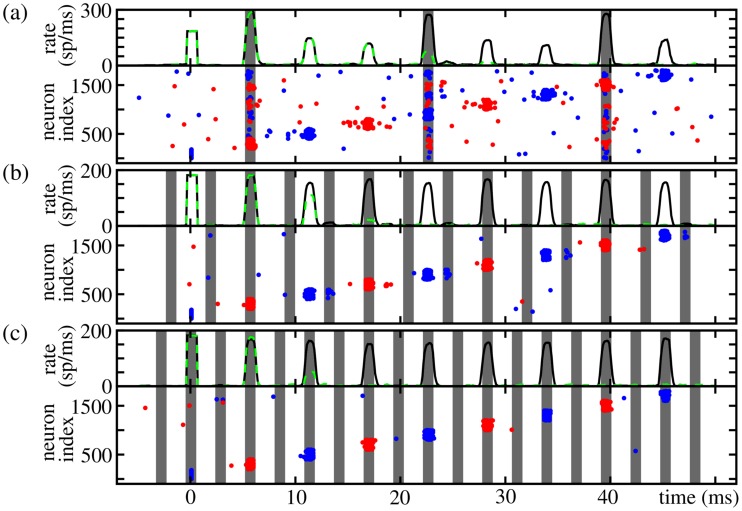
Examples of resonance in isolated FFNs with non-additive coupling (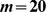, 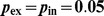, 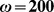). The ratio between the stimulation frequency 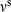 and the natural propagation frequency 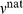 is rational: (a) 
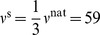
Hz, (b) 
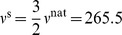
Hz and (c) 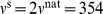
Hz. The gray areas indicate the time interval in which the external oscillations may contribute to the generation of somatic spikes. At 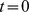 synchronous activity is induced in the first layer. The upper panels show the spiking rate of neurons of the FFN in the presence of external oscillations (black solid). The firing rates for identical networks, where the oscillatory input stops at 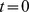 are shown for comparison (green dashed). The lower panels show the spiking activity of the first nine layers (odd layers: red, even layers: blue). Other parameters are (a–c) 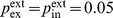, 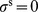
ms, 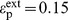
nS, 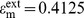 and (a) 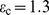
nS, 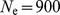, (b) 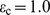
nS, 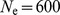 and (c) 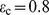
nS, 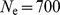.

With changing 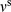, we observe multiple resonance peaks for setups where the ratio of 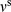 and 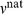 is rational, 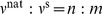 for some small integers 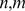. The arrival of the input from every 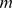
th external oscillation coincides with the arrival of the synchronous pulse from the preceding layer at every 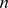
th group. Examples are shown in Fig. 7 for frequency ratios 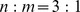 (the propagation at every third layer is supported by the external input), 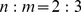 (the propagation at every second layer is supported by the external input from every third oscillation) and 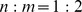 (every second oscillatory input coincides with the arrival of the synchronous pulse from the preceding layer). We remark that the sub-harmonic resonances are less prominent than the main resonance frequency, however, they can nonetheless enable oscillation-induced signal transmission even in systems where the oscillation frequency is small compared to the natural propagation frequency (cf. for example Fig. 7a).

Near the resonance frequencies the propagation frequency 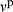 locks to the stimulation frequency 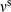 (cf. Fig. 6c gray areas). If the stimulation frequency increases above the resonance frequencies, synchrony propagation breaks down: Due to non-zero synaptic delay, initiation time of a dendritic spike and rise-time of the excitatory postsynaptic potential, there is a minimal time interval a signal needs to propagate from one layer to another. Thus, if the external stimulation frequency becomes too large, the inputs from the preceding layer arrive too late, i.e., outside the dendritic integration window 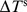, and therefore the additional inputs do not support propagation of synchrony as described above.

We only observe frequency lockings for small integers 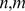. The number 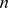 counts the (minimal) number of layers a signal has to propagate in the absence of external simulations as the propagation of synchrony is supported by the oscillatory input only for every 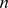
th layer. For large 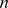, however, the signal either propagates even in the absence of additional inputs (i.e., there is no need for supporting the signal propagation) or it has decayed after 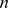 layers and cannot be stabilized by external inputs. Large 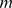 imply high stimulation frequencies, and with increasing stimulation frequency the external input becomes more and more stationary in the sense that additional (oscillatory) inputs are delivered to the neurons of the FFN all the time. A propagation of synchrony may be enabled, but the signal does not lock to the stimulation frequency anymore (cf. Fig. 6c).

Above we demonstrated how oscillations can support signal transmission in FFNs with homogenous delays. As shown in the subsequent sections, we observe the same resonance phenomena equally prominent in FFNs with distributed delays, even if the delay distributions are broad.

We also remark that we can describe the emergence of oscillation supported propagation of synchrony using methods introduced in [30], [31]. In Supporting Material S1 Text we provide a simplified, analytically tractable model by describing the dynamics in terms of probabilistic threshold units. In particular, we derive an analytical expression for the minimal amplitude of the oscillatory input, 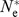, for which robust signal propagation is possible and compare the analytical predictions with numerical simulations (cf. Supporting Material S2 Text).

### Selecting transmission pathways by resonance

Networks with non-additive coupling exhibit resonance to oscillatory signals and this provides the possibility of specifically activating FFNs with different resonance frequencies. As we demonstrate below such resonant signal transmission establishes a mechanism to read out signals encoded in the structure of a recurrent network.

In how far do the results for pure feed-forward structures without recurrent connectivity can be generalized to recurrent systems as relevant for biological neural circuits? The main difference between isolated FFNs and recurrent FFNs is the emergence of a projection of the synchronous activity to all neurons of the network, not only to the neurons of the layer following the currently active one. For additively coupled networks this projection (similar to balanced oscillatory input) shifts the range of coupling strengths 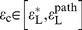
(5)for which a persistent propagation of synchrony is possible to larger connection strengths. The length of the interval, however, is unchanged (for details see Supporting Material Text S2). For non-additively coupled networks, the critical connectivity 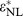 is largely unaffected, but with more and more prominent recurrent connections the pathological threshold 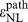 decreases. For moderate recurrent connection strengths propagation of synchrony can be induced by oscillations also in recurrent networks without causing pathological activity; though if the connections are too large activity might spread not only from one layer to the next, but might propagate over the whole network (‘synfire explosion’ activity, [25], [26], [53]). We investigate and discuss such recurrent systems in detail in Supporting Material S2 Text.

The main resonance frequency in non-additively coupled FFNs is given by the natural propagation frequency 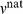. This frequency, however, is determined by the average time 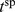 an arriving synchronous input at a given layer needs to trigger a somatic spike and the average synaptic delay 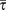, 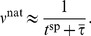
(6)


**Figure 8:**
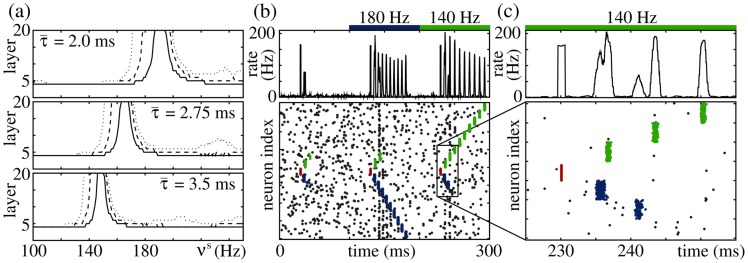
Activation of specific signal transmissions in FFNs with different resonance frequencies. (a) With increasing average coupling delays 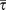 (distribution width 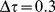
ms) resonance peaks (isolated FFN; 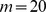, 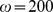, 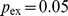, 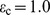
nS) are shifted to lower frequencies (cf. Equation 6). The panels show up to which layer a synchronous pulse propagates in the presence of balanced oscillations (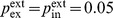, 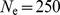, 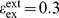
nS, 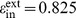
nS, 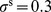
ms). The width of the resonance peaks increases with increasing size of the dendritic integration window (solid: 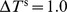
ms, dashed: 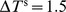
ms, dotted: 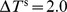
ms). (b) Raster plot of the spiking activity of a recurrent network (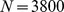, 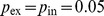, 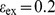
nS, 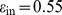
nS) which contains two FFNs (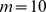, 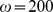, 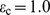
nS) which share the initial layer. Both FFNs have different average coupling delays (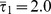
ms and 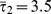
ms; 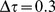
ms) and thus different resonance frequencies (cf. panel a); for the remaining connections the average coupling delays is 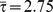
ms. Whereas a synchronous pulse extinguishes after a few layers in the absence of oscillations (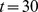
ms), it may propagate along the layers of one FFN or the other depending on the stimulation frequency (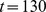
ms and 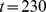
ms; 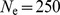, 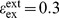
nS, 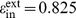
nS, 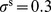
ms). Panel (c) is a close-up view of the raster plot shown in (b).

We illustrate this dependency in Fig. 8a indicating the resonance peaks for different 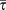. Here, the coupling delays 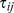 between neurons of successive layers are drawn uniformly from an interval of length 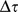 centered at 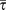, 
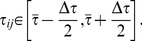
(7)


With increasing 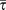, the natural propagation frequency and thus the resonance peaks are shifted to smaller frequencies.

The width of the resonance peak is determined by the temporal spread of the propagating synchronous pulse itself, the temporal spread of the oscillatory inputs (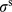; cf. also Supporting Material S1 Text) and the width of the dendritic integration window 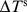. In particular, the width of the resonance peaks increases with increasing 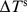 as shown in Fig. 8a.

The existence of separated resonance peaks provides the possibility to specifically activate different signal transmission routes by oscillations of suitable frequencies. As a simple example consider a recurrent network containing two FFNs (cf. Fig. 8b,c). The coupling delays between neurons of successive layers of the first FFN are centered at 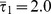
ms, the coupling delays between neurons of successive layers of the second FFN are centered at 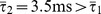. As before, the feed-forward couplings 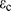 in both FFNs are too weak to enable a robust propagation of synchrony in the absence of external oscillations (cf. Fig. 8b). Yet, external oscillations fitting to the resonance frequencies of the FFNs may enable robust propagation in one of the FFNs without activating the other. The close-up view in Fig. 8c shows that indeed the propagation in both FFNs occur with different propagating frequencies.

### Heterogeneous conduction delays

So far we considered networks with homogeneous or narrow delay distribution. However, heterogeneous delays provide a desynchronizing force to propagating synchronous signals. Here, we investigate the robustness of oscillation-induced signal propagation with respect to heterogeneous coupling delays. We show that even in networks with broad delay distributions external oscillations support signal propagation.

Starting with a homogenous delay distribution, i.e., all delays 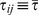, we study broadened ones in the following. More precisely, we draw the the conduction delays from a log-normal distribution, i.e., the probability density function is given by [54]

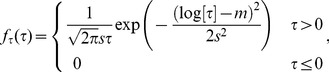
(8)where 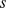 and 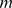 are the parameters of the probability distribution. To keep the resonance frequencies comparable, we keep the mode 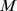, i.e., the maximum of the probability distribution (8), constant with increasing distribution width parameter 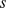. For given 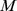 and 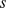, the parameter 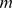 of the probability distribution (8) is given by [54]

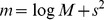
(9)and thus Equation (8) reads



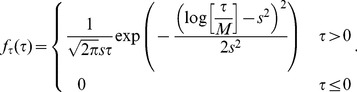
(10)The standard deviation of the distribution is given by 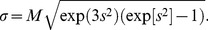
(11)


**Figure 9:**
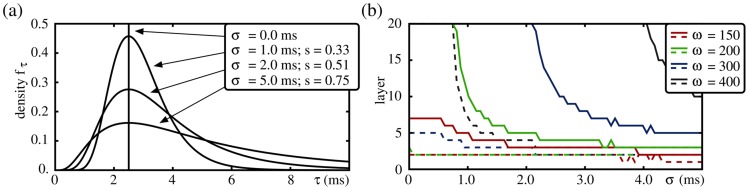
Signal propagation in FFNs with broad delay distribution. (a) Probability density function (10) of log-normal delay distribution with mode 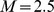
ms and different standard deviations 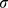 (cf. also Equation 11). (b) The panel shows up to which layer a synchronous pulse propagates in the presence (solid lines) and in the absence (dashed lines) of balanced oscillations for different layer sizes 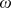 (color code). The network setup is the same as in Fig. 4 (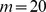, 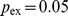, 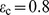
nS; with external oscillation parameters: 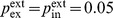, 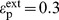
nS, 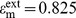
nS, 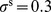
nS, 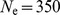, 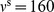
Hz). With increasing width of the delay distribution, the inputs from one layer to the following layer become more and more desynchronized, and thus signals propagate over fewer and fewer layers. However, by increasing the layer size oscillation-induced signal propagation is possible, even for very broad delay distributions. For further explanation see text.

In Fig. 9a we show the log-normal distribution for fixed 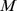 and different 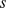 and 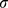.

We consider signal propagation in FFNs in the presence of balanced oscillations. Synchronous pulses may propagate along the layers, if the summed input from the external oscillation and the previous layer is strong enough to excite sufficiently many neurons to spike. However, the dendritic integration window 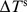 is small, typically in the order of a few milliseconds. Only inputs arriving simultaneously within this time interval can contribute to the generation of dendritic spikes, and thus may elicit subsequent somatic spikes. By increasing the width of the delay distribution, the arrival times of the inputs from the previous layer become more and more distributed. Consequently, the number of spikes arriving simultaneously with the external spikes, i.e., within a time interval 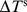 centered at the expected arrival times of the external synchronous pulses, decreases — thus, the effective number of inputs decreases (cf. also Supporting Material Text S1). However, this decrease might be compensated by, e.g., larger layer sizes 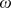. As an example, we illustrate in Fig. 9B that an FFN with a layer size of 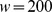 neurons (green line) can tolerate heterogeneous delay distributions with a standard deviation up to 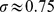
ms (same network setup as in Fig. 4b,d and 6c,d). In a similar network with increased layer sizes an oscillation-induced propagation of synchrony is possible for substantially broader delay distributions (e.g., for 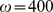 up to 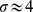
ms).

Oscillation-induced signal transmission can take place if the total expected input within the relevant time window 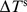 is sufficiently large. Therefore both the width 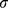 of the delay distribution and the layer size 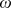 influence the width of the resonance peaks. With increasing 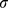 the arrival times of the spikes from the previous layer become more distributed, and the total number of spikes within a time interval 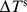 decreases.

**Figure 10:**
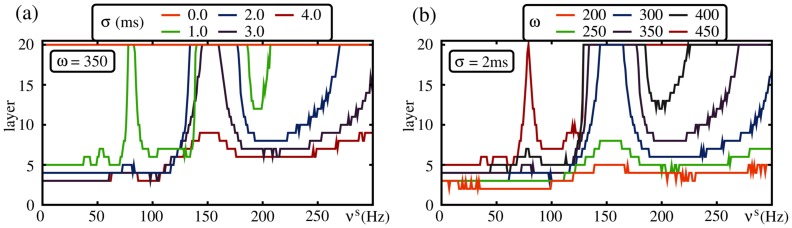
Resonances in FFNs with broad delay distribution (same network setup as in Fig. 9). The panels show until which layer a synchronous pulse successfully propagate versus the stimulation frequency 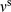. In (a) the layer size is fixed (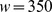) and the width 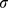 of the delay distribution is varied. Here, for heterogeneous coupling delays (orange) a synchronous signal propagates for all stimulation frequencies (and even in the absence of external stimulations). With increasing 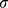 the fraction of frequencies for which a robust signal propagation is possible decreases, and for sufficiently large 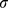 no robust signal propagation is possible anymore (red). In (b) the width of the delay distribution is fixed (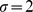
ms) and the layer size 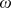 is varied. Here, for small 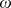 robust signal propagation is not possible (independent of the stimulation frequency), however, with increasing layer size the fraction of stimulation frequencies which enable a robust signal propagation increases. For further explanation see text.

We illustrate the effect of an increasing width of delay distribution in Fig. 10a: Starting with a setup where a synchronous signal can propagate over all layers for homogeneous coupling even in the absence of external oscillations, an increase of the width of the delay distribution results in the formation of resonance peaks. The arriving inputs become more and more distributed and therefore signal propagation is only possible if the input from the previous layer is supported by external oscillations. If the delay distribution becomes broader, the frequency bands which enable oscillation-induced signal transmission become narrower, and eventually for sufficiently large 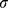 a robust signal transmission is not possible anymore.

Similarly, for a given width 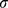 of the delay distribution an increase of the layer size 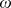 may enable oscillation-induced signal transmission and cause the formation of resonance peaks (cf. Fig. 10b). With increasing 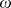 the total number of potential inputs from the previous layer (and thus also the number of potential inputs within the relevant time window of length 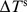) increases. If this number becomes sufficiently large, robust propagation of synchronous pulses is enabled.

We conclude that oscillation-induced signal propagation in FFNs is possible even if the delay distribution is broad, and that heterogeneities in the delays can be compensated by increased layer sizes.

We remark that heterogeneous weights (in contrast to heterogeneous delays) do not constitute a desynchronizing force in networks with nonlinear dendritic interactions: The spike latency 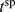 (and thus the propagation frequency) is only weakly affected by the coupling strength (cf. Fig. 3). Thus, if the input is sufficient to elicit dendritic spikes, the timing of the consecutive somatic spike (if triggered) does not depend on the input strength from the previous layer or the external input - only the timing of presynaptic inputs is important.

### Propagation in hippopcampal-like network structures

We have demonstrated that oscillation-induced signal transmission is present in systems with heterogeneous coupling delays. In the previous section we studied the influence of inhomogeneities in a rather general setting. In this section we consider a specific example: We employ a delay distribution as expected for subnetworks in the hippocampus. In this area, spike patterns generated during exploration are replayed during sleep [14], [15], [55], [56], accompanied by high-frequency network oscillations [57]–[59]. The replay has been hypothesized to be realized by local feed-forward structures [13], [14], [16], [60], possible supported by dendritic sodium spikes [30], [31], [41] which have been prominently found in the hippocampus [29], [43], [46], [61]. In the following we show that oscillations in hippocampal-like network structures indeed support signal transmission. Importantly, the expected resonance frequencies quantitatively agree with the oscillation frequencies observed in neurophysiological experiments.

We assume that the delays are a function of the distance between the presynaptic and the postsynaptic neuron. Further, we take variations of the dendritic conduction time into account. In general, the total conduction delay can be decomposed into two contributions, 
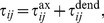
(12)(i) the axonal delay, i.e., the time interval between the presynaptic spike and the onset of the synaptic transmission, and (ii) the dendritic delay, i.e., the time delay between the onset of the synaptic transmission and the onset of the postsynaptic (somatic) response. The axonal conduction delays are proportional to the distance between the presynaptic and postsynaptic neuron. For simplicity, we assume that the neurons are distributed uniformly on a quadratic patch with side length 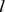. Thus,
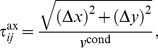
(13)where 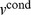 is the axonal conduction velocity and 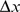 and 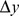 are the absolut distances between two neurons in the horizontal and vertical direction with the probability density function
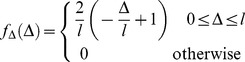
(14)for 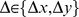. The dendritic conduction delays are drawn uniformly from the interval
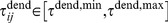
(15)and account for the variability in the distance between the synaptic contact sites and the soma.

As an example, we consider the recurrent connections in the hippocampal region CA1. Here, the range of local axonal interconnections is estimated to be in the order of 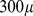
m [62], [63]; in some direction connections extending over 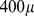
m or more are found [62]–[65]. The axonal conduction delay 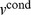 in the hippocampus is measured to be in the range of 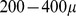
m
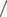
ms [66], [67], for numerical simulation we assume 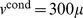
m/ms in the middle of the biologically plausible parameter range. Further, we assume the variation in the dendritic conduction delays to be in the interval 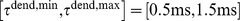 in agreement with experimental data [68]–[70].

**Figure 11:**
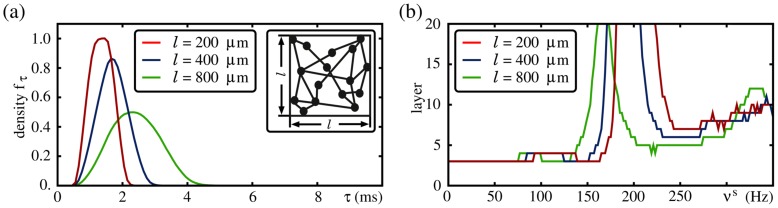
Signal propagation in hippocampal-like networks. (a) Probability density function for delay distributions of neurons on a quadratic patch with side length 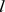. The conduction delay is composed of the distance-dependent axonal delay and the uniformly distributed dendritic delay (for details see Equations (12) – (15) and explaining text). (b) The panel shows up to which layer a synchronous pulse propagates along an FFN with the delay distribution taken from (a) in the presence of balanced oscillations for different patch sizes 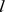. The network setup is the same as in Fig. 9. With increasing patch size 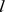, and thus increasing connection lengths, the resonance frequencies are shifted to lower values. For further discussion see text.

In Fig. 11a we show the resulting probability density functions for different patch sizes 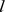. With increasing side length 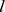 the probability distributions become broader and the peak of the distribution is shifted to larger delays. As shown in Fig. 11b synchronous pulses may propagate in FFNs in the presence of external oscillations. We observe resonances as before (cf. Fig. 6), and the resonance frequencies are shifted to smaller frequencies with increasing patch size 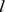.

Interestingly, the oscillation frequencies accompanying replay in the hippocampus are larger in area CA1 than in the more globally connected region CA3 [57], [71], [72]. We hypothesize that the existence of long range connection in CA3 (and therefore an effectively increased patch size) cause lower resonance frequencies. The observed oscillations during replay might therefore be optimized for the specific region and support the replay of spike patterns encoded in weak FFNs.

The oscillations themselves might be generated by global network oscillations based on dendritic spikes [50], by highly connected nodes (so-called hub-neurons) which are a prominent feature of networks with broad degree distribution [41], by interneuron network oscillations [57], [73], [74], or by avalanches of spikes propagating in a network of axons coupled by axo-axonal gap junctions [75]–[77].

## Discussion

Reliable and controlled transmission of signals is considered essential for computation in cortical networks. Propagation of synchronous activity along layered feed-forward networks may be one important way to realize such transmission [19], [21], [23]. Starting with a random recurrent network, feed-forward structures are assumed to be formed in a “training phase” previous to the recall of the learned sequences by, e.g., spike time dependent plasticity [78]–[80]. Moreover, propagating synchronous pulses are a candidate for generating precisely timed spike patterns in the millisecond range as observed in various neurophysiological studies (e.g., [81]–[84]).

Robust propagation, however, typically requires a highly prominent feed-forward anatomy, either in the sense of densely connected layers of neurons [25]–[27] or strongly increased connection strengths between neurons of successive layers (compared to remaining connections of the network) [28]. Such prominent structures are experimentally not observed.

In previous articles we have shown that fast dendritic spikes can support signal transmission in the form of propagation of synchrony [30], [31]. They specifically amplify activity that is synchronous, and thus enable a robust propagation in networks with moderate feed-forward anatomy. In this article we demonstrated that the presence of background oscillations can relax this requirement even further by supporting the propagating signal by additional external inputs. These additional inputs excite the neurons of the network (including the current target layer of the propagating synchronous pulse) and therefore enable a robust propagation with less inputs from the preceding layer. As a consequence robust signal transmission may emerge in networks with weaker couplings between the layers of the feed-forward network.

Such weaker structures, where the differences between feed-forward connections and remaining recurrent couplings are smaller, can be formed faster by synaptic plasticity (assuming a constant plasticity rate), i.e., the process of creating (and reconfiguring) information pathways is simplified. Alternatively, the background oscillations can enable robust signal transmission in feed-forward networks with reduced layer size (while keeping the coupling strengths fixed). We may expect that this leads to an increase in the storage capacity of recurrent networks, because a reduced number of “memory-encoding” neurons is required for reliable signal propagation.

We remark that the mechanism of oscillation-induced signal transmission is related to the idea of “communication through coherence” [33], where the information flow between neural groups is enabled by coherent rhythmic modulation in the neural excitability in the different sub-networks. Similarly, in our approach the oscillatory input excites the neurons (and, even more importantly, the non-linear dendrites of the neurons) of the local network, and therefore acts as a “clock” enabling the successful propagation of synchronous pulses in the local network.

Experimental data suggests that there is a balance between excitatory and inhibitory input to single neurons in cortical networks during spontaneous and sensory-evoked activity [85]–[87]. We therefore considered external oscillatory input which is composed of an excitatory as well as an inhibitory component. We find that for additively coupled networks, only unbalanced external inputs that cause a net depolarization, support propagation of synchrony. Further, this support does not depend on the oscillatory nature of the input and could equally well be established by a temporally constant input current with the strength of the temporal mean input.

In contrast, for networks with non-additive couplings the ratio of the excitatory and inhibitory input is less important. In these networks propagation of synchrony is mainly mediated by dendritic spikes, which are elicited if the excitatory input within a short time interval exceeds the dendritic threshold [29], [42]–[44]. Further, inhibition fails to suppress the generation of such dendritic spikes [46] and thus even inputs with a net hyperpolarizating effect support signal propagation. Due to the short dendritic integration window the timing of the external input is important, and thus only oscillatory inputs of a suitable frequency range can facilitate the propagation of synchrony. Whenever the ratio of the stimulating frequency and the “natural” propagation frequency of the feed-forward network is rational, resonances and locking patterns emerge. The resonance frequencies themselves are determined by the average conduction delays between the neurons of the FFN. This provides a mechanism to selectively activate different signaling pathways by oscillations of suitable frequency.

If either the synaptic couplings or the oscillatory inputs are too strong, synchronous activity may spread over the entire network, generating a large scale synchronous population burst and a subsequent phase of refractoriness. The occurrence of such pathological activity states which transiently silences the network can terminate the induced propagating signal and therefore hinder signal transmission. These observations may be relevant for understanding the neurological implications of epileptic-like seizures.

For clarity of presentation, we first demonstrated the effect of oscillation-induced propagation of synchrony for FFNs with homogenous or relatively narrow delay distributions. In biological neural circuits, the distribution of delays might be substantially broader. One might expect that this may blur out signals and hinder their reliable transmission. However, in networks with nonlinear dendrites, for the generation of dendritic spikes (and consecutive somatic spikes) inputs from both, the previous layer and the oscillatory network are needed. Therefore, broad delay distributions only decrease the “effective” layer size, i.e. the fraction of inputs from the previous layer which can arrive within the relevant time interval to support spike generation. As a consequence FFNs with broad delay distribution require a moderately increased layer size, but the general mechanism of oscillation-induced signal transmission is unchanged.

In this article we considered oscillatory input arriving from an external source. For clarity, we separated the local (signal processing) network and the oscillation-generating network to study the impact of oscillations. We note that there are no conceptual differences if we consider networks, in which such oscillations arise from the embedding network itself. For example, we have recently shown that in networks with a broad distribution of synaptic connections moderate network oscillations which are suited to support signal transmission naturally emerge [41]: So-called hub-neurons (higher than average connected neurons) can echo the propagating synchronous signal, start to oscillate and therefore provide an oscillatory, supporting feedback. As another example intrinsic network oscillations can emerge due to recurrent inhibition or the excitatory-inhibitory loop [88], [89]. Oscillation-supported signal transmission might also arise from network intrinsic responsivity modulations such as sub-threshold membrane potential oscillations in resonator-type neurons [90], if they are synchronized and sufficiently strong to depolarize the dendritic compartments in a rhythmic way. Furthermore, network level resonances [91] may support propagation of synchrony.

Dendritic spikes are prominently found in, e.g., the hippocampus (cf. [29], [43], [46], [61] and others). In this cortical area spike patterns observed during spatial exploration are replayed during sleep or resting phases (e.g., [14], [15], [55], [56]). Interestingly, this replay is accompanied by high-frequency oscillations in the range of up to 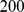 Hz [57],[58],[71]. We estimate the distribution of conductance delays for recurrent connections in the hippocampal areas CA1/CA3, and show that the expected resonance frequencies for the support of synchrony propagation agree quantitatively with the frequencies observed in neurophysiological experiments. This suggests that the high-frequency oscillations may contribute to the stabilization of the replay of spike patterns in the hippocampus.

Our choice of parameters, including that of (average) conduction delays, is guided by neurophysiological observations in the hippocampus. However, in other cortical systems substantially larger delays have been reported (see, e.g., [92] for an overview). Because the natural propagation frequency decreases with increasing conduction delays, this suggests that the mechanism of oscillation-induced signal transmission is not restricted to high-frequency oscillations as present in the hippocampus. Furthermore, oscillations can stabilize signal transmission for stimulation frequencies where the ratio of natural propagation frequency and stimulation frequency is rational. Therefore oscillation-induced signal transmission can be enabled by stimulation with frequencies substantially smaller than the natural propagation frequency. For example, only every second or third synchronous pulse might be supported by the oscillatory input (cf. Fig. 7). The widths of these sub-harmonic resonances are smaller than the main resonance peak (around 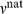), however, we have shown that they can enable oscillation-induced signal transmission even if the oscillation frequencies are small compared to the natural propagation frequency.

Finally, we emphasize that the occurrence of the identified mechanism of signal transmission by oscillation-induced propagation of synchrony need not be restricted to information processing in neural networks. In Supporting Material S1 Text, we derive a simplified, analytically tractable model describing the network activity in terms of probabilistic threshold units. Its analysis reveals that the main prerequisite for oscillation-induced signal transmission is the threshold-like processing of inputs of the single elements in the network. We may therefore expect that the mechanism also plays a role in other networks of sharply nonlinear threshold units. Networks of such units describe a variety of real-world phenomena, like the transmission of rate activities in neural networks (McCullogh-Pitts model, e.g., [59], [93]), (failure) cascades in social, supply or communication networks (e.g., [94], [95]), or signaling in gene and protein networks (threshold Boolean networks, e.g., [96].

## Methods

In this section we briefly introduce the neuron model and system setup. A complete list of standard neuron and model parameters is provided in the last subsection.

### Neuron model

We consider networks of neurons of the integrate-and-fire type [97]. Single neurons interact by sending and receiving action potentials (spikes). The state of neuron 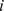 is described by its membrane potential 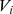 and its temporal dynamics are determined by 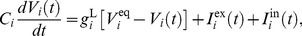
(16)where 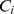 is the membrane capacity, 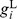 is the leak conductance and 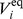 is the equilibrium potential. 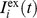 and 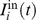 are currents arising from excitatory and inhibitory inputs, respectively. Whenever the membrane potential 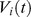 exceeds the spiking threshold 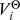 at some time 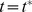, a spike is sent to the post-synaptic neurons 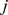, where it arrives after a delay time 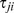. The sending neuron's potential is reset to 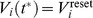, and the neuron is refractory for a time period 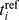, i.e., 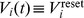 for 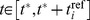. Simulation results were obtained using the simulation software NEST [98].

### Linear (additive) coupling

The effects of the synaptic inputs on postsynaptic neurons are modeled by transient conductance changes. Denoting the reversal potentials of excitatory and inhibitory currents by 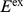 and 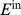, the input currents to neuron 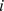 arising from synaptic inputs from other neurons of the network are given by 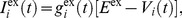
(17)

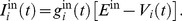
(18)



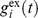
 and 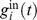 are linear superpositions of single responses, 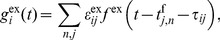
(19)

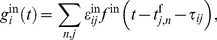
(20)where 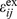 and 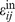 denote the excitatory and inhibitory coupling strength from neuron 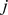 to neuron 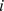 and 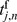 is the 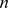
th spiking time of neuron 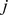. 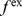 and 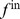 specify the time course of the synaptic conductance change given by the difference of two exponentials [97] with time constants 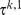 and 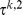,
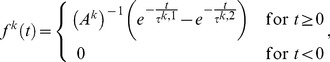
(21)for 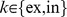 describing the effect of an excitatory and inhibitory input, respectively, that is received at time 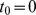. The normalization constant
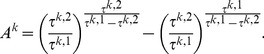
(22)is chosen such that the peak conductance 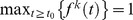. Throughout this article, we denote the strength of a synaptic connection by the value of the peak conductance, i.e., a single input of strength 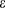 causes a conductance change 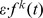.

We note that, to keep the model as simple as possible, we did not incorporate any saturation in the linear model. This is in contrast to the model with nonlinear dendrites (see below), since a dendrite becomes refractory after generation of a dendritic spike.

### Non-linear (non-additive) coupling

Besides linear summation of inputs (as described above), we consider nonlinear amplification of synchronous inputs mediated by fast dendritic spikes. These have been found in single neuron experiments (e.g., [29], [42]–[44]) and introduced in recent models of neural networks [30], [41], [45], [50], [99]. The amplification is based upon dendritic action potentials which generate a strong depolarization in the soma. Here, three properties are of particular interest: (i) The amplification is very sensitive to input synchrony (relevant time window 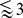 milliseconds), (ii) the peak of the depolarization in the postsynaptic neuron (pEPSP) is reached a certain time interval after stimulation with only sub-millisecond jitter and (iii) with increasing stimulation strength the amplitude of the pEPSP saturates.

We model the contribution of such dendritic spikes to the neuronal input as follows (see also [30], [50]): We augment the neurons with an additional nonlinear dendrite. Inputs that arrive at the linear dendrite are processed as described above. Inputs on the nonlinear dendrite also cause a conductance change as described above, but additional depolarizations of the membrane potential mimicking the effect of a dendritic spike may be generated. If the total excitatory input to a nonlinear dendrite within a time interval 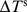 exceeds a certain threshold 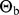, a current pulse is initiated which takes effect on the membrane potential after a delay time 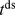. To account for the experimentally observed saturation of the somatic depolarization caused by dendritic spikes we limit the maximal conductance change within a time interval 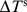 to 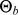 and model the current pulse in a phenomenological approach such that the depolarization caused by a suprathreshold input, 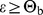, resembles the characteristics and time course of the depolarization observed in single neuron experiments (cf. [29]). More precisely, the current pulse is described by the sum of three exponential functions, 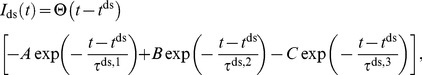
(23)with positive prefactors 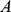, 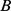, 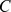 and decay time constants 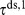, 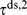 and 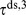 which are chosen such that the somatic depolarization fits experimental data. After initiation of such a current pulse the (nonlinear) dendrite becomes refractory for a time period 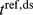 and does not transmit spikes within the refractory time period. This refractoriness yields the experimentally observed saturation for inputs exceeding the dendritic threshold.

We note that for the generation of a dendritic spike only the excitatory inputs are considered. Consequently, in accordance with recent experimental findings, inhibition fails to suppress fast dendritic sodium spikes. However, the probability that a somatic spike is initiated by a dendritic one might be reduced by hyperpolarization of the soma [46] (cf. also [41]).

### Network setup

We investigate sparsely, randomly connected recurrent networks and study the propagation of synchrony in naturally occurring feed-forward subnetworks (FFNs). “Naturally occurring” here means that the feed-forward structures are present as part of a recurrent network and are not generated by, e.g., adding feed-forward connections. However, they are highlighted by moderately increased excitatory connections.

We denote the total number of neurons in the recurrent network by 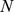. The network itself constitutes an Erdös-Rényi random graph: A directed excitatory synaptic connection between any pair of neurons exists with probability 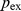. Inhibition in recurrent networks is usually assumed to be mediated by a population of inhibitory neurons (interneurons). Spiking of excitatory neurons causes a response of inhibitory neurons which in turn project an inhibitory input to the excitatory neurons. Here, we simplify this inhibitory feed-back mechanism and assume that the spiking of neurons, additionally to the excitatory input on the postsynaptic neurons, have an inhibitory effect: An inhibitory connection between any pair of neurons exists with probability 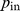. We remark that there might exist an inhibitory and excitatory connection between two neurons. However, these cases are rare due to the sparsity of the considered networks (typically 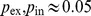). The simplification of the inhibitory feed-back loop eases the analytical treatment, but is not crucial for the effect of oscillation induced propagation of synchrony as discussed later on (cf. also [41]).

For clarity of presentation coupling strengths are assumed homogeneous; excitatory connections have strength 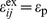, the strength of inhibitory connections is denoted by 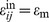. We choose the ratio between inhibitory and excitatory connection strengths, 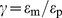, such that the peaks of single excitatory and inhibitory postsynaptic potentials measured at resting membrane potential are of equal amplitude.

We define FFNs by assigning neurons randomly to 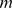 groups of 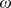 neurons each, where each neuron belongs to one group at most. These groups constitute the layers of the FFN. By construction, the connectivity between neurons of successive groups of the FFN statistically equals the overall connectivity. To enable propagation of synchrony, we increase the strengths of the already existing excitatory connections between neurons of successive layers; this connection strength is denoted by 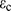.

For clarity of presentation, in the first part of the article we investigate the influence of oscillations on propagating synchrony in isolated FFNs. Here, only excitatory connections between neurons of successive layers are present, i.e., 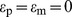, but 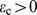. However, recurrent connections (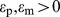) do not change the results qualitatively. We comprehensively study recurrent FFNs and discuss differences to isolated FFNs in Supporting Material S2 Text.

### Detecting propagation pulses

We evaluate up to which layer a synchronous pulse propagates in the FFN by considering the signal-to-noise ratio (SNR):

After a synchronous pulse is initiated in the first layer (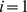) at time 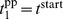, we determine for the following layers 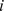, 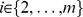, how many neurons have spiked within a time window of length 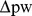 lagging behind the synchronous pulse in the previous layer (centered at 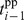) by a temporal shift 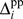. The temporal shift 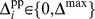 is chosen after simulation such that the number of spikes 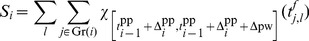
(24)becomes maximal. Here 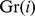 are the indices of neurons of group 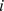, 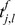 is the 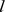
th firing time of neuron 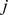, and 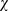 denotes the characteristic function. Starting with 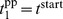, the following 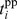 are determined by first evaluating 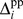 according to Equation (24), and then defining 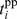 as the mean of all spikes contained in the interval 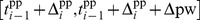.

Further, we determine the noise level 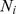 in each layer 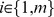 by measuring the probability 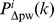 to find 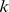 spikes from neurons of group 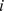 in a time window 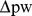 during a control time interval 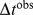 in which no synchronous activity is induced (an external oscillatory input is present, if applicable). The noise level 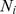 is then given by the minimal value satisfying 
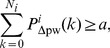
(25)with constant 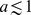. Finally, we denote the propagation up to the 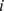
th layer as successful if the SNR is larger than a constant 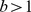,



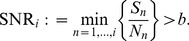
(26)This means, in particular, that we can distinguish the background (spontaneous) activity from the transmitted signal in all layers 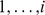.

### Homogeneous neuronal background

In the ground state of balanced networks [2], [3] single neurons fire irregularly and their spiking activity is approximately described by Poissonian spike trains [4], [100], [101]. In addition to inputs from the recurrent network each neuron receives inputs from remote networks, and we emulate this influence by independent excitatory and inhibitory (Poissonian) spike trains. We denote the rates by 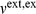 and 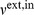 and the strength of single spikes (peak conductances) by 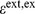 and 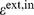, respectively. Similarly to the recurrent connections, we assume the external input to be balanced, such that the total input is balanced as well. As a consequence, the neurons are in a fluctuation-driven regime, and in the absence of synchrony the neurons spike asynchronously and irregularly and their output spike trains resemble Poissonian spike trains themselves.

### Background oscillations

In this article we study the impact of neuronal oscillations on the ability of recurrent networks to propagate synchronous signals. Oscillatory input may arise from oscillations in other circuits or within the local network itself.

**Figure 12:**
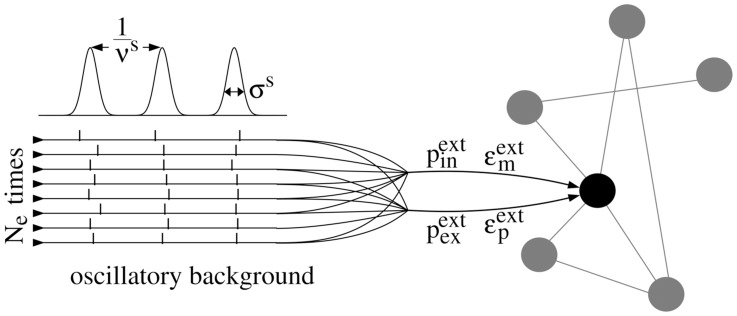
Schematic illustration of oscillatory background input. Oscillatory input is generated by a (virtual) population of 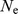 neurons which spike once during each oscillation period of length 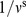. The actual spiking times are drawn from a Gaussian distribution. At each neuron in the network, each spike causes an excitatory input of strength 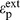 with probability 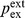 and an inhibitory input of strength 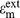 with probability 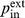. Additionally to the oscillatory input, neuron receive inputs from recurrent connections and Poissonian spike trains which are not displayed in the fig.

To systematically investigate the influence of oscillations on synchrony propagation in a controlled way, we emulate such oscillations by excitatory and inhibitory inputs generated by a ‘virtual’ population of 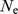 neurons that spike with a mean frequency 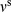. Within each oscillation period 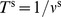, 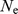 spike times are drawn from a Gaussian distribution centered at 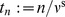 (for the 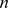
th oscillation, 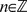) with standard deviation 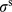. Each of these spikes causes an excitatory input of strength 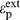 with probability 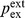 and an inhibitory input of strength 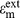 with probability 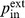 to each neuron of the recurrent network (cf. Fig. 12).

Here and in the following the term “balanced oscillations” refers to oscillatory input for which excitatory inputs and inhibitory inputs cause postsynaptic potentials of equal amplitude if the average excitatory inputs exceed the inhibitory inputs or vice versa, we denote such inputs as “unbalanced oscillations”.

Whereas unbalanced oscillations induce a net depolarization or hyperpolarization of the neurons in the network, balanced oscillations maintain the balance between excitation and inhibition, and are thus expected to change the average membrane potential in the ground state only weakly. However, they may influence the effective time constant of the neurons as discussed in the Results Section (cf. also [51], [52]).

The aim of the article is to understand the influence of the oscillatory nature of the input on propagating synchrony, and resonances between signal propagation and input oscillations. We discuss balanced oscillations and unbalanced oscillations separately.

### Standard parameters

Throughout the article (for simplicity) we consider a homogeneous neuron population. The single neuron parameters are 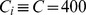
pF, 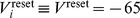
mV, 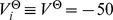
mV, 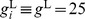
nS, 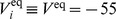
mV and 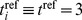
ms [72], [102] for all 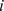.

The time constants of the excitatory conductances (AMPA) are 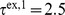
ms and 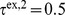
ms [103], [104]. For simplicity we assume the same time constants for inhibitory conductances (GABA_A_), 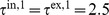
ms and 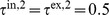
ms. The reversal potentials are 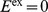
mV and 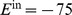
mV [72], [97]. To obtain balanced recurrent (and external oscillatory) inputs, the ratio 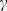 between excitatory and inhibitory couplings is chosen such that the peaks of single excitatory and inhibitory postsynaptic potentials equal each other when the inputs are received at resting membrane potential, i.e., 
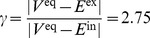
(27)for standard neuron parameters.

We consider sparsely connected networks (standard connection probability 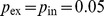) with homogenous coupling delays 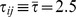
ms in the first part of the article, and with heterogeneous coupling delay distribution in the second part. For the latter, the underlying distribution and parameters are stated in the corresponding sections.

Each neuron receives excitatory and inhibitory Poissonian spike trains with rates 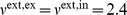
kHz. Single inputs have strength 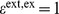
nS and 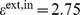
nS, respectively.

The parameters of the dendritic spike current are chosen according to single neuron experiments [29], [42]–[44], 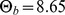
nS, 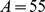
nA, 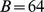
nA, 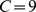
nA, 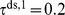
ms, 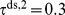
ms, 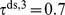
ms and 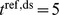
ms (cf. also, [30], [50]). The standard value for the length of the dendritic integration window is 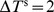
ms; in the last part of the article it is varied as indicated.

For the detection of propagating synchronous signals, we considered time windows of length 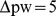
ms, and considered time lags between successive synchronous pulses up to 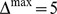
ms. The noise level is determined during an observation interval 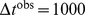
ms, we further set the constant for defining the chance level to 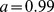 and require a minimal SNR of 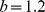.

## Supporting Information

S1 Text **Analytical considerations.**
(PDF)

S2 Text **Synchrony propagation in recurrent FFNs.**
(PDF)
