# Supplementary material for: Oscillation-Induced Signal Transmission and Gating in Neural Circuits
Source: PLoS Comput Biol. 2014 Dec 11;10(12):e1003940. doi: 10.1371/journal.pcbi.1003940 (PMC4263355; doi:10.1371/journal.pcbi.1003940)
Supplement: S1 Text — Analytical considerations. (PDF) [file pcbi.1003940.s001.pdf]

Supporting Material Text S1: Analytical considerations

Sven Jahnke[1−3], Raoul-Martin Memmesheimer[4], and Marc Timme[1−3]
[1] *Network Dynamics, Max Planck Institute for Dynamics & Self-Organization (MPIDS), 37077 Göttingen, Germany,*
[2] *Bernstein Center for Computational Neuroscience (BCCN), 37077 Göttingen, Germany,*
[3] *Institute for Nonlinear Dynamics, Fakultät für Physik, Georg-August-Universität Göttingen, and*
[4] *Donders Institute, Department for Neuroinformatics, Radboud University, Nijmegen, Netherlands.*

Supplemental material accompanying the article
Oscillation-induced signal transmission and gating in neural circuits

Under which conditions do oscillations induce a transition from a regime of non-robust to robust synchrony propagation? In particular, what is the (minimal) amplitude or the degree of synchrony required that allow for robust signal propagation? To answer this question, we investigate the emergence of oscillation-supported propagation in FFNs with non-additive couplings analytically. We employ a self-consistency approach (cf. also methods introduced in [30, 31, 45]) to derive an approximation of the iterated map for the average size of a synchronous pulse that propagates along the layers of an FFN. In particular, we find a scaling law for the amplitude of the external oscillations that enable stable propagation as a function of the system parameters and the dendritic nonlinearity.

Synchronous spiking of neurons in some layer causes a synchronous input to the neurons of the next layer. In the presence of oscillations of suitable frequency, e.g., $\nu^{\mathrm{s}} \approx \nu^{\mathrm{nat}}$, this input may be supported by inputs from the external oscillations. Then the total excitatory input

$$I = I_{\mathrm{e}} + I_{\mathrm{c}} \tag{S1.1}$$

is the sum of inputs arising from external oscillations, I_{e}, and from the preceding layer, I_{c}. In networks with non-additive coupling, the spiking probability p^{sp} due to a synchronous input I below the dendritic threshold Θ_{b} is typically much smaller than due to a suprathreshold input (cf. Figure 2a). We thus assume that only neurons that receive a suprathreshold input ($I > \Theta_{\mathrm{b}}$) generate a spike with fixed probability p^*, i.e.,

$$p^{\mathrm{sp}}(I) := \begin{cases} p^* & \text{if } I \geq \Theta_{\mathrm{b}} \\ 0 & \text{if } I < \Theta_{\mathrm{b}} \end{cases}. \tag{S1.2}$$

Thus, neurons process synchronous signals like simple threshold units, i.e., they generate no response for subthreshold inputs and a fixed response for suprathreshold inputs. For clarity of presentation, we assume that the firing probability p^* is fixed. In general it might be reduced by inhibitory input, but the extension is straightforward and leads to similar results (cf. also [41]).

The timing of somatic spikes initiated by dendritic spikes is highly precise, i.e., the temporal distribution of somatic spikes triggered by dendritic ones is very narrow (cf. Figure 3a), in the sub-millisecond range (cf. also [29]). In particular, the jitter in time is typically much smaller than the dendritic integration window ΔT^{s}. This let us assume that a synchronous pulse packet in one layer causes synchronous spiking within a time interval smaller than ΔT^{s} in the next layer and so on.

In the following we calculate the probability density function $f_I(I)$ for the total excitatory input I to the neurons of a given layer conditioned on (i) the number of synchronously spiking neurons in the previous layer, g^{in}, and (ii) the amplitude of external oscillations, N_{e}. Then, the average number of synchronously spiking neurons in the considered layer is

$$g^{\mathrm{out}} = \omega \int_0^\infty p^{\mathrm{sp}}(I) f_I(I|g^{\mathrm{in}}, N_{\mathrm{e}}) dI \tag{S1.3}$$

$$= \omega p^* \int_{\Theta_{\mathrm{b}}}^\infty f_I(I|g^{\mathrm{in}}, N_{\mathrm{e}}) dI. \tag{S1.4}$$

First we consider the input from the previous layer. Given the random topology of the FFN, the probability that a neuron receives exactly k (out of the maximal number g^{in}) inputs is binomially distributed,

$$p(k) = \binom{g^{\mathrm{in}}}{k} (p_{\mathrm{ex}})^k (1 - p_{\mathrm{ex}})^{g^{\mathrm{in}} - k}. \tag{S1.5}$$

For a sufficiently large number g^{in} of neurons participating in the synchronous pulses, we can approximate the binomial distribution (S1.5) by a Gaussian distribution and thus the excitatory synchronous input follows

$$I_{\mathrm{c}} = k\varepsilon_{\mathrm{c}} \sim \mathcal{N}\left(\mu_{\mathrm{c}}, \sigma_{\mathrm{c}}^2\right) \tag{S1.6}$$

with mean

$$\mu_{\mathrm{c}} = \varepsilon_{\mathrm{c}} g^{\mathrm{in}} p_{\mathrm{ex}} \tag{S1.7}$$

and standard deviation

$$\sigma_{\mathrm{c}} = \varepsilon_{\mathrm{c}} \sqrt{g^{\mathrm{in}} p_{\mathrm{ex}} (1 - p_{\mathrm{ex}})}. \tag{S1.8}$$

Likewise, the number of excitatory inputs l each neuron receives within one oscillation period from the external (virtual) neuron population is binomially distributed, $l \sim B(N_{\mathrm{e}}, p_{\mathrm{ex}}^{\mathrm{ext}})$. The arrival times are drawn from a

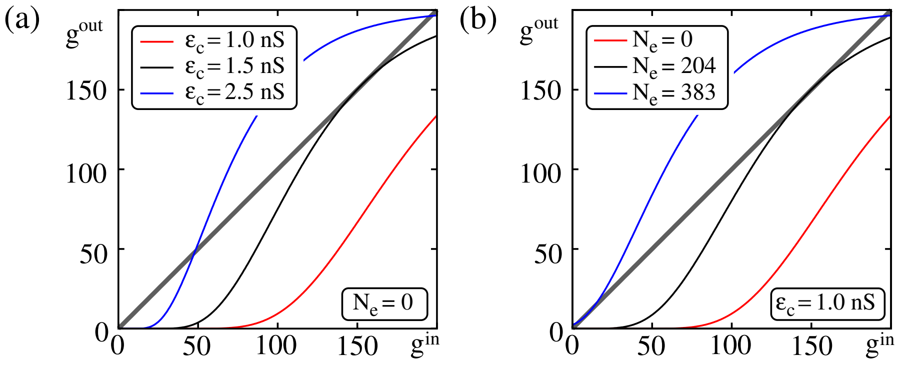

FIG. S1.1: **Map yielding the temporal evolution of the average size of a synchronous pulse in an FFN with non-additive coupling** (cf. Equation S1.17; $\omega = 200$, $p_{\mathrm{ex}} = 0.05$, $\Theta_{\mathrm{b}} = 8.65$nS). (a) $N_e = 0$ (absence of external oscillations), different colors indicate different coupling strength ε_{c}. (b) The coupling strength $\varepsilon_{\mathrm{c}} = 1.0$nS is fixed and external oscillations ($p_{\mathrm{ex}}^{\mathrm{ext}} = 0.05$, $\varepsilon_{\mathrm{p}}^{\mathrm{ext}} = 0.3$nS, $\sigma^{\mathrm{s}} = 0$ms) are present, different colors indicate different N_{e}. With increasing (a) connection strength ε_{c} or increasing (b) oscillation amplitude N_{e}, two fixed points emerge by a tangent bifurcation. This bifurcation point marks the transition from a regime where no propagation is possible to a regime where persistent propagation of synchrony can be achieved (cf. also Figure 2b,c).

Gaussian distribution with standard deviation σ^{s}. We assume that propagation of synchrony in the FFN and the external oscillations are in-phase. Then for $\sigma^{\mathrm{s}} > 0$ the fraction

$$p_{\Delta T^{\mathrm{s}}} = \int_{-\frac{\Delta T^{\mathrm{s}}}{2}}^{\frac{\Delta T^{\mathrm{s}}}{2}} \frac{1}{\sqrt{2\pi}\sigma^{\mathrm{s}}} \exp\left[-\frac{1}{2}\left(\frac{\tau}{\sigma^{\mathrm{s}}}\right)^2\right] d\tau \qquad \text{(S1.9)}$$

$$= \mathrm{Erf}\left(\frac{\Delta T^{\mathrm{s}}}{\sqrt{8}\sigma^{\mathrm{s}}}\right) \qquad \text{(S1.10)}$$

of the additional inputs arrive within the dendritic integration window ΔT^{s} and can support the generation of dendritic spikes. For $\sigma^{\mathrm{s}} = 0$, all inputs are received synchronously and thus $p_{\Delta T^{\mathrm{s}}} = 1$; for non-zero σ^{s} the effective size of the external oscillation (i.e., the effective average number of neurons that may contribute to the generation of dendritic spikes) is

$$N_{\mathrm{e}}^{\mathrm{eff}} = p_{\Delta T^{\mathrm{s}}} N_{\mathrm{e}}, \qquad \text{(S1.11)}$$

and the number of excitatory inputs from the oscillatory neuron population is distributed according to $l \sim B\left(N_e^{\mathrm{eff}}, p_{\mathrm{ex}}^{\mathrm{ext}}\right)$.

For sufficiently large $N_{\mathrm{e}}^{\mathrm{eff}}$, one can again use a Gaussian approximation which yields

$$I_{\mathrm{e}} \sim \mathcal{N}\left(\mu_{\mathrm{e}}, \sigma_{\mathrm{e}}^2\right) \qquad \text{(S1.12)}$$

with

$$\mu_{\mathrm{e}} = \varepsilon_{\mathrm{p}}^{\mathrm{ext}} N_{\mathrm{e}}^{\mathrm{eff}} p_{\mathrm{ex}}^{\mathrm{ext}} \quad \text{and} \quad \sigma_{\mathrm{e}} = \varepsilon_{\mathrm{p}}^{\mathrm{ext}} \sqrt{N_{\mathrm{e}}^{\mathrm{eff}} p_{\mathrm{ex}}^{\mathrm{ext}} \left(1 - p_{\mathrm{ex}}^{\mathrm{ext}}\right)}. \qquad \text{(S1.13)}$$

The sum of the inputs I_{e} and I_{c} is then also approximately Gaussian distributed,

$$I = I_{\mathrm{e}} + I_{\mathrm{c}} \sim \mathcal{N}\left(\mu, \sigma^2\right), \qquad \text{(S1.14)}$$

with mean $\mu = \mu_{\mathrm{e}} + \mu_{\mathrm{c}}$ and variance $\sigma^2 = \sigma_{\mathrm{e}}^2 + \sigma_{\mathrm{c}}^2$, i.e.,

$$\mu = \varepsilon_{\mathrm{p}}^{\mathrm{ext}} N_{\mathrm{e}}^{\mathrm{eff}} p_{\mathrm{ex}}^{\mathrm{ext}} + \varepsilon_{\mathrm{c}} g^{\mathrm{in}} p_{\mathrm{ex}} \qquad \text{(S1.15)}$$

and

$$\sigma = \sqrt{\left(\varepsilon_{\mathrm{p}}^{\mathrm{ext}}\right)^2 N_{\mathrm{e}}^{\mathrm{eff}} p_{\mathrm{ex}}^{\mathrm{ext}} \left(1 - p_{\mathrm{ex}}^{\mathrm{ext}}\right) + \varepsilon_{\mathrm{c}}^2 g^{\mathrm{in}} p_{\mathrm{ex}} \left(1 - p_{\mathrm{ex}}\right)}. \qquad \text{(S1.16)}$$

Using the distribution (S1.14) of I allows us to specify the iterated map for the average size of a synchronous pulse according to Equation (S1.4),

$$g^{\mathrm{out}} = \frac{\omega p^*}{2}\left(1 + \mathrm{Erf}\left[\frac{\mu - \Theta_{\mathrm{b}}}{\sqrt{2}\sigma}\right]\right), \qquad \text{(S1.17)}$$

where the size of the initial pulse packet g^{in} appears as argument of μ and σ (see Equations S1.15 and S1.16).

The fixed points $G^* = g^{\mathrm{out}} = g^{\mathrm{in}}$ of Equation (S1.17) determine the stability of the propagation of a synchronous pulse. With increasing coupling strength two fixed points emerge via a tangent bifurcation (Figure S1.1a; cf. also Figure 2b,c), and external oscillations have a similar effect (Figure S1.1b). This transition enables robust propagation of synchrony, and the external oscillations thus reduce the critical connection strength $\varepsilon_{\mathrm{NL}}^*$ (i.e., the minimal coupling strength for which robust signal propagation is possible).

For a given network setup, $N_{\mathrm{e}} = N_{\mathrm{e}}^*$ specifies the minimal size of the external oscillation which enables stable propagation of synchrony. It can be found by numerically determining the bifurcation point of Equation (S1.17). Additionally, one can derive a scaling law for N_{e}^* based on two observations:

1. In the absence of external oscillations ($N_{\mathrm{e}} = 0$), the position of the bifurcation point of Equation (S1.17) depends on the coupling strength ε_{c} and

the dendritic threshold Θ_b only via the quotient

$$\kappa := \frac{\Theta_\mathrm{b}}{\varepsilon_\mathrm{c}}, \tag{S1.18}$$

which is the number of spikes from the preceding layer that are needed to elicit a dendritic spike. Equation (S1.17) reads

$$g^\mathrm{out} = \frac{\omega p^*}{2}\left(1 + \mathrm{Erf}\left[\frac{g^\mathrm{in}p_\mathrm{ex} - \kappa}{\sqrt{2g^\mathrm{in}p_\mathrm{ex}\left(1-p_\mathrm{ex}\right)}}\right]\right). \tag{S1.19}$$

For a given network setup, the connection probability p_ex, group size ω and spiking probability p^* (which is determined by the ground state and the parameters of the dendritic spike) are fixed. Thus the bifurcation point where the fixed points $G_1^* = G_2^* = g^\mathrm{out} = g^\mathrm{in}$ appear by a tangent bifurcation, depends solely on κ (the only unknown quantity). Consequently, there is some $\kappa^* = \kappa$ specifying this bifurcation point, i.e., the transition point from non-propagating to propagating regime depends just on the number of spikes necessary to elicit a dendritic spike. The actual value κ^* can be found either by numerical simulation of the system, numerical solution of Equation (S1.19) or by the analytical methods introduced in [30, 31].

2. The main influence of external oscillatory inputs is an effective reduction of the dendritic threshold Θ_b, such that the properties of the system described above can be approximated by a network without external oscillatory input, but with a reduced dendritic threshold $\Theta_\mathrm{b}^\mathrm{eff} < \Theta_\mathrm{b}$: In the setups considered the additional oscillatory input contributes to the generation of dendritic spikes, but the main contribution arises from the input arriving from the previous layer (the signal to be propagated), i.e., $\mu_\mathrm{e} < \mu_\mathrm{c}$. Moreover, the feed-forward connections ε_c are enhanced compared to the remaining excitatory couplings, $\varepsilon_\mathrm{p}^\mathrm{ext} < \varepsilon_\mathrm{c}$. Thus the total variation of the input $\sigma = \sigma_\mathrm{e}^2 + \sigma_\mathrm{c}^2$ (cf. Equation S1.16) is dominated by the contribution σ_c^2 of the input from the previous layer,

$$\varepsilon_\mathrm{p}^\mathrm{ext}\mu_e\left(1-p_\mathrm{ex}^\mathrm{ext}\right) < \varepsilon_\mathrm{p}\mu_\mathrm{c}\left(1-p_\mathrm{ex}\right) \tag{S1.20}$$

$$\sigma_e^2 < \sigma_\mathrm{c}^2. \tag{S1.21}$$

In particular for $\varepsilon_\mathrm{p}^\mathrm{ext} \ll \varepsilon_\mathrm{c}$ the contribution of the external inputs to the total variation of the input becomes negligible, i.e., $\sigma_\mathrm{e}^2 \ll \sigma_\mathrm{c}^2$, and the argument of the error function in Equation (S1.17) simplifies to

$$\frac{\mu - \Theta_\mathrm{b}}{\sqrt{2}\sigma} = \frac{\mu_\mathrm{c} + \mu_\mathrm{e} - \Theta_\mathrm{b}}{\sqrt{2\left(\sigma_\mathrm{c}^2 + \sigma_\mathrm{e}^2\right)}} \approx \frac{\mu_\mathrm{c} - \Theta_\mathrm{b}^\mathrm{eff}}{\sqrt{2}\sigma_\mathrm{c}} \tag{S1.22}$$

where we defined the effective dendritic threshold

$$\Theta_\mathrm{b}^\mathrm{eff} := \Theta_\mathrm{b} - \mu_\mathrm{e}. \tag{S1.23}$$

The above observations indicate that the bifurcation point is found for some constant

$$\kappa^* = \frac{\Theta_\mathrm{b}^\mathrm{eff}}{\varepsilon_\mathrm{c}} = \frac{\Theta_\mathrm{b} - \mu_\mathrm{e}}{\varepsilon_\mathrm{c}}, \tag{S1.24}$$

such that the minimal size of the external oscillations N_e^*, which enables propagation of synchrony, is given by (using Equations S1.13, S1.11 and S1.10)

$$N_e^* = \mathrm{Erf}\left[\frac{\Delta T^\mathrm{s}}{\sqrt{8}\sigma^\mathrm{s}}\right]^{-1}\frac{\Theta_b - \varepsilon_\mathrm{c}\kappa^*}{\varepsilon_\mathrm{p}^\mathrm{ext}p_\mathrm{ex}^\mathrm{ext}}. \tag{S1.25}$$

Equation (S1.25) indicates that N_e^* changes linearly with the coupling strength ε_c (cf. Figure 4 and 5). Further it is inversely proportional to the coupling strength between the external oscillatory population and the neurons of the FFN, $N_\mathrm{e}^* \propto 1/\varepsilon_\mathrm{p}^\mathrm{ext}$, and the dependence of N_e^* on the temporal spread σ^s of the external oscillations is determined by the prefactor $1/p_{\Delta T^\mathrm{s}}$.

The above results are derived for isolated FFNs. However, we show and discuss in Supporting Material Text S2 that the results hold in good approximation also for FFNs that are part of recurrent networks.
