# Supplementary material for: Oscillation-Induced Signal Transmission and Gating in Neural Circuits
Source: PLoS Comput Biol. 2014 Dec 11;10(12):e1003940. doi: 10.1371/journal.pcbi.1003940 (PMC4263355; doi:10.1371/journal.pcbi.1003940)
Supplement: S2 Text — Synchrony propagation in recurrent FFNs. (PDF) [file pcbi.1003940.s002.pdf]

# Supporting Material Text S2: Synchrony propagation in recurrent FFNs

Sven Jahnke[1–3], Raoul-Martin Memmesheimer[4], and Marc Timme[1–3]
[1]*Network Dynamics, Max Planck Institute for Dynamics & Self-Organization (MPIDS), 37077 Göttingen, Germany,*
[2]*Bernstein Center for Computational Neuroscience (BCCN), 37077 Göttingen, Germany,*
[3]*Institute for Nonlinear Dynamics, Fakultät für Physik, Georg-August-Universität Göttingen, and*
[4]*Donders Institute, Department for Neuroinformatics, Radboud University, Nijmegen, Netherlands.*

Supplemental material accompanying the article
*Oscillation-induced signal transmission and gating in neural circuits*

For clarity of presentation, in this article we focus mainly on isolated FFNs (i.e., $\varepsilon_{\mathrm{m}} = \varepsilon_{\mathrm{p}} = 0$; only feed-forward connection are present). However, in this Supporting Material we show that FFNs which are part of a random recurrent network show qualitatively the same dynamics as isolated ones. We consider recurrent FFNs where all neurons of the network are assigned to be a member of exactly one layer, i.e., $N = m\omega$. In the first part we consider propagation of synchrony in front of a homogeneous background activity, and afterwards, in the second part, we study the impact of external oscillations. We investigate the influence of network parameters, discuss the differences to isolated FFNs and compare the results of network simulations with the analytical predictions presented in Supporting Material Text S1.

### Homogenous background activity

What is the impact of recurrent connections on propagation of synchrony? Do recurrent connections within the FFN alter its propagation efficiency?

To gain some insight into the dynamics of recurrent FFNs, we start with isolated FFNs ($\varepsilon_{\mathrm{p}} = \varepsilon_{\mathrm{m}} = 0$, as before) and gradually increase the recurrent coupling strengths $\varepsilon_{\mathrm{p}}$ and $\varepsilon_{\mathrm{m}}$. We keep the ratio of the coupling strengths $\varepsilon_{\mathrm{p}}$ and $\varepsilon_{\mathrm{m}}$ balanced (i.e., $\varepsilon_{\mathrm{m}} = \gamma \cdot \varepsilon_{\mathrm{p}}$, cf. Methods Section), such that the mean additional input to each neuron arising from recurrent connections is approximately zero and the network remains in the balanced state [2–4].

For networks with linear dendritic interactions, the critical connection strength $\varepsilon_{\mathrm{L}}^{*}$ (the minimal coupling strength $\varepsilon_{\mathrm{c}}$ for which a robust propagation of synchrony is possible) as well as the pathological connection strength $\varepsilon_{\mathrm{L}}^{\mathrm{path}}$ (the maximal coupling strength $\varepsilon_{\mathrm{c}}$ for which a non-pathological propagation of synchrony is possible) increases with increasing recurrent connection strength $\varepsilon_{\mathrm{p}}$ and $\varepsilon_{\mathrm{m}}$. However, the length of the interval

$$\left[\varepsilon_{\mathrm{L}}^{*}, \varepsilon_{\mathrm{L}}^{\mathrm{path}}\right] \tag{S2.1}$$

is only weakly affected (cf. Figure S2.1b).We note that the additional input arising from the projection of the synchronous pulse in one layer to the whole network (instead of only to the following layer) is similar to the input originating from balanced external oscillations (cf. also Figure 4) and this additional balanced inputs decrease the excitability of the neurons by lowering the effective membrane time constant (as discussed in the Results Section of the article) [51, 52].

In contrast, in networks with nonlinear dendritic interactions, where propagation of synchrony is mainly mediated by dendritic spikes, recurrent connections influence the critical connection strength $\varepsilon_{\mathrm{NL}}^{*}$ only weakly (cf. Figure S2.1c): In principle, the additional inputs arising from recurrent connections support the generation of dendritic spikes as additional excitatory inputs effectively lower the dendritic threshold $\Theta_{\mathrm{b}}$, and therewith decrease $\varepsilon_{\mathrm{NL}}^{*}$ (compare also the analytical considerations in Supporting Material Text S1). However, in the ground-state the neurons of the recurrent network spike asynchronously with a low rate and therefore the additional excitatory input to each neuron within the dendritic integration window $\Delta T^{\mathrm{s}}$ is small compared to the dendritic threshold $\Theta_{\mathrm{b}}$.

However, recurrent connections decrease the pathological connection strength $\varepsilon_{\mathrm{NL}}^{\mathrm{path}}$, above which propagation of synchrony causes pathological network states: In recurrent FFNs all neurons, not only the neurons belonging to one specific layer, receive synchronous inputs if a synchronous pulse packet propagates along the layers of an FFN. Thus each neuron which is not member of the currently active layer receives an additional (compared to the isolated FFN) input, the projection of the synchronous activity. The average strength of the excitatory part of the input during persistent propagation is given by

$$I^{\mathrm{add}} = g^{*} p_{\mathrm{ex}} \varepsilon_{\mathrm{p}} \lessapprox \omega p_{\mathrm{ex}} \varepsilon_{\mathrm{p}}, \tag{S2.2}$$

where $g^{*}$ denotes the average size of the propagating pulse and $g^{*} \approx G_{2}^{*}$ with the stable fixed point $G_{2}^{*}$ of the iterated map (S1.17) (cf. also Equation 2). $I^{\mathrm{add}}$ effectively decreases the dendritic threshold, i.e., even for neurons that do not belong to the next layer the amount of synaptic input needed to elicit a dendritic spike is reduced by propagation of synchrony. This can become detrimental for information processing: In combination with inputs arising from spontaneous activity the additional input may induce synchronous spiking in currently non-active layers and the synchronous pulse starts to spread over the whole network causing pathological activity ('synfire explosion',cf.[26]). Thus recurrent connections within the

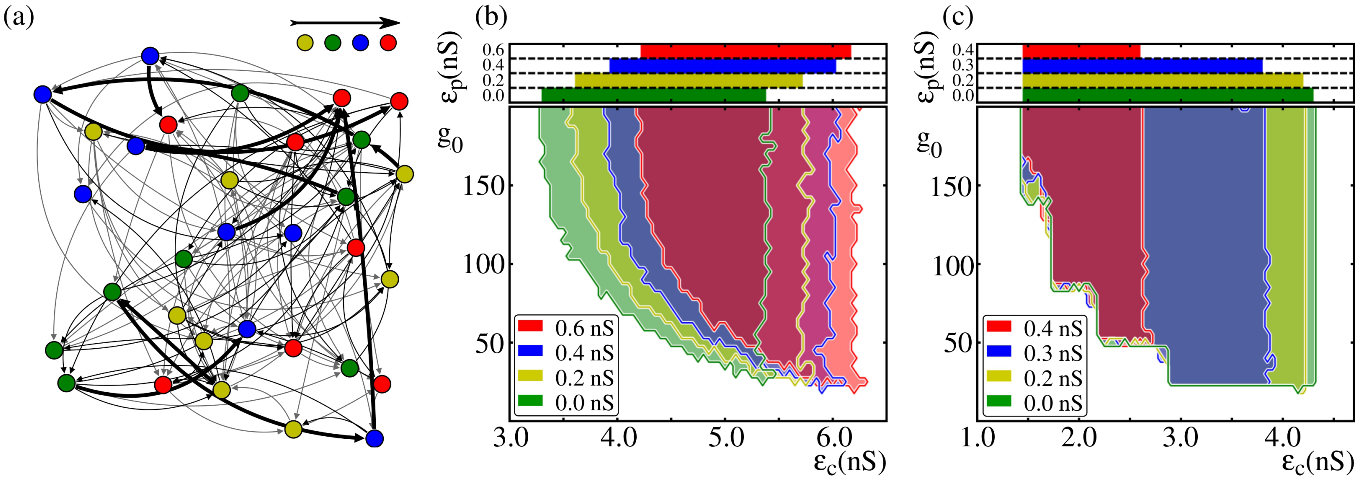


FIG. S2.1: **Propagation of synchrony in embedded FFNs.** (a) Scheme of the network setup: We show an example network of 32 neurons that are assigned to 4 consecutive layers (yellow→green→blue→red). The arrows indicate connections between neurons (excitatory: black; inhibitory: gray), their thickness highlights the connection strength (thin arrows: $\varepsilon_p$ and $\varepsilon_m$; thick arrows: feed-forward couplings $\varepsilon_c$, with increased strength). The connectivity between neurons of successive layers equals the overall connectivity. (b,c) Upper panel: Illustration of the range of coupling strengths $\varepsilon_c$ between the layers of the FFN that allow for a robust and non-pathological propagation of synchrony in recurrent networks ($N = 3000$, $m = 15$, $\omega = 200$, $p_{ex} = p_{in} = 0.05$) with (b) linear and (c) nonlinear dendritic interactions for different recurrent connection strengths $\varepsilon_p$ (color coded; $\varepsilon_m = 2.75\varepsilon_p$). The lower panel shows the region of successful propagation (horizontal axis: size of the initial synchronous pulse, $g_0$; vertical axis: feed-forward coupling strength $\varepsilon_c$).

FFN decrease the length of the interval

$$\left[\varepsilon^*_{NL}, \varepsilon^{path}_{NL}\right] \quad (S2.3)$$

of coupling strengths for which a non-pathological propagation of synchrony is possible (cf. Figure S2.1c).

### Background oscillations

External oscillations can induce robust synchrony propagation in networks with recurrent connections. The underlying mechanism is the same as in isolated chains. The amplitude $N^{path}_e$ of external oscillation above which the system enters pathological dynamics is reduced by recurrent connections. The critical amplitude $N^*_e$ for which the transition from non-propagating to oscillation induced propagation of synchrony occurs, however, is largely unaffected. Moreover, the analytical considerations for $N^*_e$ derived in Supporting Material Text S1 are in good approximations also for recurrent FFNs.

In this section we consider recurrent FFNs as introduced above and assume that the connectivity between the external oscillating (virtual) neuron population and the neurons of the network is statistically identical to the recurrent connectivity in the network itself, i.e., $\varepsilon_p = \varepsilon^{ext}_p$, $\varepsilon_m = \varepsilon^{ext}_m$, $p_{ex} = p^{ext}_{ex}$ and $p_{in} = p^{ext}_{in}$.

According to Equation (S1.25) we expect a linear relationship between the excitatory feed-forward coupling strength $\varepsilon_c$ and the amplitude $N^*_e$. Indeed, we observe such a relation in isolated (Figure 4b,d) as well as in recurrent FFNs (Figure S2.2a). The impact of recurrent connections on $N^*_e$ is negligible (as discussed above) and Equation (S1.25) well predicts the scaling of $N^*_e$ (dashed line in Figure S2.2a).

However, the presence of recurrent connections lowers the threshold for pathological activity, $N^{path}_e$: In absence of recurrent connections ($\varepsilon_p = \varepsilon_m = 0$) an external oscillation of size $N_e \geq N^{path}_e$ causes pathological activity (cf. Figure 4b,d). Here, in the presence of recurrent connections all neurons ‘feel’ the propagating synchronous signal through recurrent projections. The recurrent input resembles an external oscillatory input of size $g^*$ (where $g^*$ is the average size of the propagating synchronous pulse packet) with coupling strengths $\varepsilon^{ext}_p = \varepsilon_p$ and $\varepsilon^{ext}_m = \varepsilon_m$. Thus, the threshold for pathological activity $N^{path}_e$ is reduced by the average size of the propagation pulse packet $g^*$, $N^{path}_e \to N^{path}_e - g^*$ as illustrated in Figure S2.2d.

Further, Equation (S1.25) indicates that $N^*_e$ is inversely proportional to the excitatory coupling strength $\varepsilon^{ext}_p$. Indeed, for small $\varepsilon^{ext}_p$ large amplitudes $N_e$ of oscillations are required to enable propagation of synchrony and with increasing $\varepsilon^{ext}_p$ smaller and smaller amplitudes of oscillations are sufficient (cf. Figure S2.2b). At the same time the threshold for pathological activity decreases: By increasing the excitatory connection strengths $\varepsilon_p$ and $\varepsilon^{ext}_p$ both (i) the impact of the projection of the propagating synchronous pulse and (ii) the impact of external oscillations increase. For sufficiently large recurrent coupling strengths the threshold for pathological activity, $N^{path}_e$, decreases below the critical oscillation amplitude, $N^{path}_e \leq N^*_e$. The sum of the projection of the

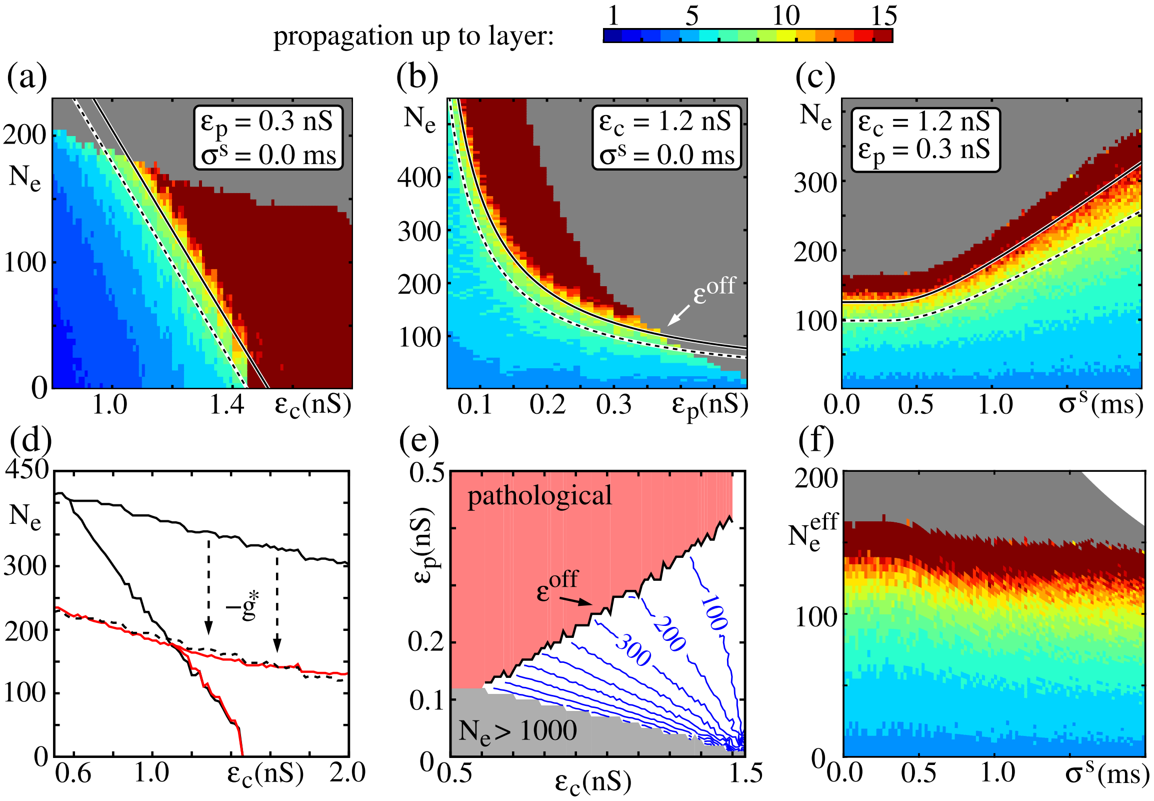


FIG. S2.2: **Oscillation induced propagation of synchrony in recurrent FFNs** ($m = 15$, $\omega = 200$, $p_{\rm ex} = p_{\rm in} = 0.05$). Panels (a-c) show up to which layer a synchronous pulse propagates in the presence of external network oscillations (cf. also Fig. 4d). The lines indicate the estimated critical amplitude $N_{\rm e}^*$, above which propagation of synchrony is enabled, derived from Equation (S1.17) (solid; numerical solution of the fixed point equation) and Equation (S1.25) (dashed). Gray areas indicate parameter sets causing pathological activity. The individual panels show the amplitude of the external oscillations vs. (a) the feed-forward connectivity $\varepsilon_{\rm c}$, (b) the recurrent connectivity $\varepsilon_{\rm p} = \varepsilon_{\rm p}^{\rm ext}$ and (c) the width of the external oscillations $\nu^{\rm s}$ (other parameters are fixed with values displayed in the insets). In panel (d) we compare $N_e^*$ and $N_e^{\rm path}$ for recurrent FFNs (red solid line; same data as in panel a) and isolated FFNs (black solid line, $\varepsilon_{\rm p}^{\rm ext} = \varepsilon_{\rm m}^{\rm ext} = 0$). The black dashed line indicates the pathological threshold $N_e^{\rm path}$ for isolated FFNs reduced by the average size $g^*$ of a (stable) propagating synchronous pulse. Panel (e) shows the region of recurrent coupling strengths, $\varepsilon_{\rm p} = \varepsilon_{\rm p}^{\rm ext}$, and feed-forward coupling strengths, $\varepsilon_{\rm c}$, for which propagation of synchrony is possible (white area) or causes pathological activity (red area). The separation line is denoted by $\varepsilon^{\rm off}$ (cf. panel b). The blue lines indicate the contour lines of $N_e^* = \{100, 200, \ldots, 900\}$. For setups where $N_e^* > 1000$ due to limitations of computing capabilities no simulations are performed (gray area). (f) Same data as in panel (c), but with rescaled (effective) size $N_e^{\rm eff}$ of external oscillations (cf. Equation S1.11).

propagating synchronous signal and the external oscillation becomes large and even spontaneous spiking activity is sufficient to trigger more and more spikes in the network and thus cause pathological activity ('synfire-explosion'). For given coupling strength $\varepsilon_{\rm c}$ between the layers of the FFN there is a maximal recurrent coupling strength $\varepsilon^{\rm off}$, such that for $\varepsilon_{\rm p} = \varepsilon_{\rm p}^{\rm ext} \geq \varepsilon^{\rm off}$, no meaningful, i.e., non-pathological, propagation of synchrony is possible (cf. Figure S2.2b). In Figure S2.2e we illustrate the region of coupling strengths ($\varepsilon_{\rm p} = \varepsilon_{\rm p}^{\rm ext}$ and $\varepsilon_{\rm c}$) for which a robust propagation of synchrony can be achieved given that an external oscillation of suitable size is present. In particular, it turns out that the maximal recurrent coupling strength $\varepsilon^{\rm off}$ depends linearly on the feed-forward coupling strength $\varepsilon_{\rm c}$ between the layers of the FFN.

Finally, Equation (S1.25) predicts that $N_e^*$ is related to the temporal width $\sigma^{\rm s}$ of the external oscillations via the factor $p_{\Delta T^{\rm s}}$ (cf. Equation S1.10). As discussed in Supporting Material Text S1 the effective size $N_e^{\rm eff}$ of the external oscillation decreases with increasing $\sigma^{\rm s}$ (cf. Equation S1.11). Consequently, the critical size $N_e^*$ and the pathological threshold $N_e^{\rm path}$ increase. However, the length of the interval

$$[N_e^*, N_e^{\rm path}]\,, \tag{S2.4}$$

i.e., the size of the interval of oscillation amplitudes that enable persistent propagation of synchrony, stays almost constant (Fig. S2.2c,f).
